# A Reproducible Hierarchical Virtual Screening Framework Integrating Scaffold-Aware Machine Learning, Ensemble Docking, and Molecular Dynamics: Application to IDO1

**DOI:** 10.1021/acs.jcim.6c00967

**Published:** 2026-05-29

**Authors:** Elisabetta Grazia Tomarchio, Rocco Buccheri, Antonio Rescifina

**Affiliations:** † Department of Drug and Health Sciences, 9298 University of Catania, Viale A. Doria 6, 95125 Catania, Italy; ‡ Department of Biomedical and Biotechnological Sciences, University of Catania, Via Santa Sofia 97, 95123 Catania, Italy

## Abstract

Indoleamine 2,3-dioxygenase 1 (IDO1) is a heme-containing enzyme implicated in cancer immune escape and remains an attractive therapeutic target despite recent clinical setbacks. We report a fully reproducible hierarchical virtual screening framework integrating scaffold-aware machine learning, ensemble docking, consensus scoring, and molecular dynamics simulations for robust prioritization of IDO1 inhibitors. A curated ChEMBL data set of IDO1 inhibitors was subjected to strict standardization, duplicate removal, and activity binarization at pChEMBL ≥6. Models were trained using scaffold-based splitting and nested cross-validation to prevent chemical series leakage. An ensemble of Random Forest, XGBoost and SVM classifiers achieved balanced predictive performance (ROC-AUC ≈0.88–0.89) with applicability domain filtering to ensure reliability. Prospective screening of FDA-approved drugs yielded 39 compounds within the applicability domain predicted as active. These were further evaluated through ensemble docking against multiple IDO1 crystal structures using GNINA with CNN rescoring. Consensus strategies were systematically benchmarked, demonstrating that best-Z-score aggregation outperformed mean, rank-based, and weighted methods in enrichment factor (EF) metrics. Two top-ranked candidates were subjected to 300 ns molecular dynamics simulations, revealing stable binding modes and persistent interactions with key catalytic residues. This study demonstrates that hierarchical integration of scaffold-aware machine learning with structure-based ensemble strategies enhances robustness and reduces false positives in virtual screening campaigns. The proposed workflow is generalizable and supports reproducible candidate prioritization in computational drug discovery. The complete implementation, including data processing, model training, and analysis steps, is provided as a fully executable Jupyter notebook available at https://github.com/rocco-b/IDO1-inhibitors-ML-and-docking-data.

## Graphical abstract

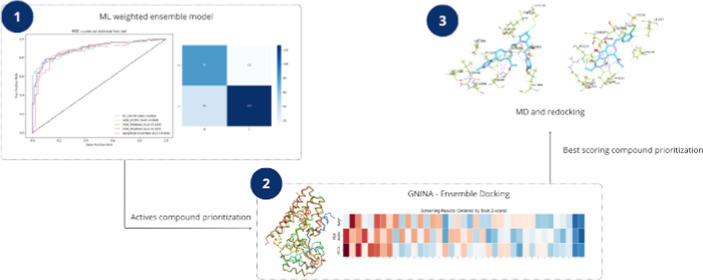

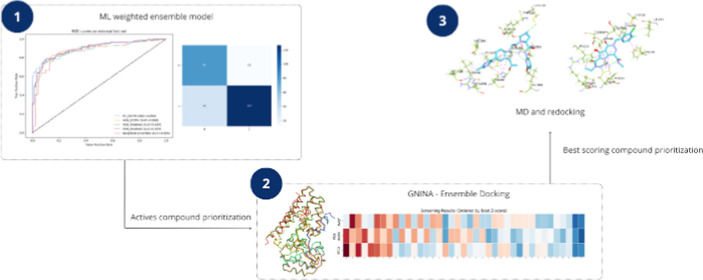

## Introduction

Computational methods play a central role in early stage drug discovery by reducing the experimental burden associated with large-scale synthesis and biological screening. Among these, quantitative structure–activity relationship (QSAR) modeling and molecular docking remain two of the most widely adopted paradigms for in silico prioritization. QSAR approaches leverage statistical and machine learning (ML) techniques to infer activity directly from molecular descriptors, whereas docking evaluates putative binding modes within a protein structure. Despite decades of development, both strategies exhibit well-recognized methodological limitations that affect robustness and generalizability. Classical QSAR models are particularly sensitive to data set composition and often overestimate performance when chemical series leakage or scaffold redundancy is not adequately controlled. Generalization beyond the training distribution remains a major concern, especially when predicting activity for structurally novel compounds.^1^ Structure-based methods face complementary challenges: conventional docking relies on rigid receptor representations and empirical scoring functions that inadequately capture the complexity of protein–ligand interactions, often resulting in limited early enrichment and high false-positive rates.^2^ These issues are exacerbated for flexible targets, where conformational heterogeneity significantly influences binding-site geometry.

Recent methodological advances have partially mitigated this issue. Ensemble-based machine learning models, combining algorithms such as Random forest (RF), extreme Gradient Boosting (XGBoost), and support vector machines (SVM), have demonstrated improved predictive stability relative to single estimators.^3^ In parallel, convolutional neural network (CNN)–enhanced scoring functions have extended docking beyond traditional empirical paradigms by learning three-dimensional interaction patterns directly from structural data.^4,5^ For example, GNINA integrates CNN-based rescoring and has been shown to outperform classical scoring functions across multiple benchmarks.^6,7^ Nevertheless, each individual methodology remains intrinsically constrained: ligand-based models are limited by their applicability domain, whereas structure-based methods are sensitive to receptor conformation selection and scoring-function bias.^8^

Integrative frameworks that combine scaffold-aware machine learning with ensemble docking and systematic consensus ranking offer a strategy to exploit the complementary strengths of ligand- and structure-based paradigms while minimizing their individual weaknesses. By coupling statistically controlled QSAR filtering with structure-based refinement across multiple receptor conformations, such hierarchical workflows can reduce false positives, improve early enrichment, and enhance mechanistic interpretability. Importantly, explicit benchmarking of consensus strategies and definition of applicability domains are essential to ensure reproducibility and avoid overestimation of predictive performance.

Indoleamine 2,3-dioxygenase 1 (IDO1) represents a relevant case study for evaluating integrated approaches. IDO1 is a heme-dependent enzyme that catalyzes the rate-limiting step of the tryptophan–kynurenine pathway and plays a central role in tumor immune evasion through tryptophan depletion and kynurenine-mediated immunosuppression.^9,10^ Although initial clinical programs targeting IDO1, including the ECHO-301/KEYNOTE-252 trial evaluating epacadostat in combination with pembrolizumab, failed to demonstrate clinical benefit,^11^ mechanistic insights have reshaped the therapeutic landscape. Distinct classes of inhibitors have been identified, including apo-IDO1 inhibitors that displace the heme cofactor and exhibit prolonged target engagement kinetics.^12,13^ Structural studies have revealed substantial conformational plasticity within the IDO1 active site, including rearrangements of flexible loops and aromatic residues that define multiple binding subsites. This conformational heterogeneity makes IDO1 a challenging target for rigid docking protocols and motivates the adoption of ensemble-based structure modeling.

Crystal structures have demonstrated that linrodostat (BMS-986205) and structurally related compounds bind to the apo form of IDO1 by displacing the heme cofactor from the catalytic pocket, thereby preventing heme rebinding and enabling sustained target engagement.^13^ This mechanism confers a distinct pharmacodynamic profile relative to classical holo-IDO1 inhibitors, as apo-binding compounds avoid direct competition with the tryptophan substrate and exhibit prolonged residence times at the enzyme. Consistent with this mechanism, apo-IDO1 inhibitors have shown enhanced cellular potency and more durable pharmacodynamic responses in vivo compared to heme-coordinating inhibitors. From a structural perspective, IDO1 poses significant challenges for computational modeling because of pronounced conformational heterogeneity within the active site. The enzyme comprises at least two partially overlapping binding regions (commonly referred to as Pockets A and B) that reorganize upon ligand binding.^14,15^ In particular, the flexible loop spanning residues 260–265 undergoes substantial positional shifts to accommodate chemically diverse scaffolds. Additionally, key residues, including Phe270, Phe214, His346, and Arg343, display side-chain rearrangements that dynamically redefine subpocket topology and interaction networks depending on ligand chemotype and heme occupancy state. This structural plasticity directly impacts pose prediction and scoring reliability, as single static receptor conformations may not adequately represent binding-competent states. Consequently, rigid single-structure docking protocols risk underestimating true binders and inflating false negatives, particularly for apo-binding chemotypes.

These structural and mechanistic considerations motivated the adoption of a hierarchical and ensemble-based computational strategy explicitly designed to address receptor flexibility and model bias. In this study, we present a reproducible hierarchical virtual screening framework for the prioritization of inhibitors within a chemically diverse space of FDA-approved compounds. The workflow integrates the following features: (i) scaffold-aware ensemble machine learning classification with applicability domain filtering to control generalization; (ii) ensemble docking against multiple IDO1 crystal structures using CNN-based rescoring to capture conformational variability; (iii) systematic benchmarking of consensus ranking strategies to mitigate structure-dependent scoring artifacts; and (iv) molecular dynamics (MD) simulations for dynamic refinement and stability assessment of top-ranked candidates (Figure 1). Rather than relying on a single predictive modality, the framework explicitly combines orthogonal information sources to enhance robustness and reduce methodological bias.

**Figure 1:**
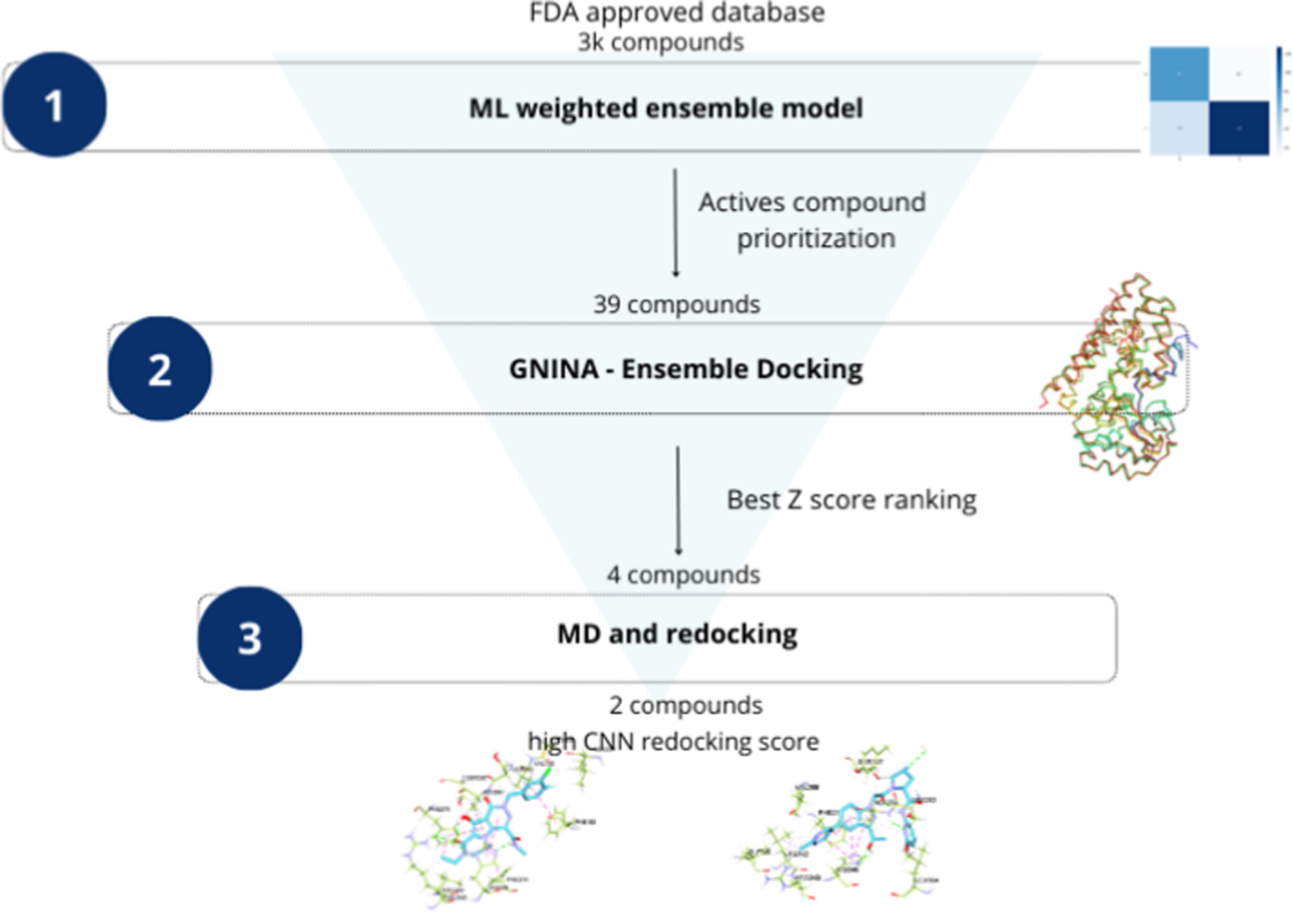
Schematic representation of the consequential workflow used in this study.

By applying this multitiered strategy to IDO1, we demonstrated how the hierarchical integration of ligand-based and structure-based methods can improve prioritization reliability for conformationally flexible targets. Beyond the specific case study presented here, the proposed workflow is modular and transferable, supporting statistically grounded and reproducible decision-making in computational drug discovery and drug repurposing campaigns.^16^

## Results and Discussion

### Scaffold-Aware Machine Learning Classification: Model Development and External Validation

Data curation was performed following established best practices for QSAR modeling to ensure reproducibility and minimize bias.^17^ After removing duplicates, inconsistent annotations, and incomplete activity records, 1277 unique compounds tested against IDO1 were retained. Activity values spanned a broad dynamic range (pChEMBL 4.00–9.92; mean 6.09), providing adequate separation between inactive and active chemotypes.

Chemical space analysis was conducted using t-distributed stochastic neighbor embedding (t-SNE) applied to Morgan fingerprints. The resulting projection (Figure 2) revealed a heterogeneous distribution of chemotypes with no evident clustering of activity labels, indicating that IDO1 inhibition was not confined to a narrow structural family. This diversity supports the suitability of a scaffold-based validation protocol and reduces the likelihood of trivial structure–activity memorization.

**Figure 2:**
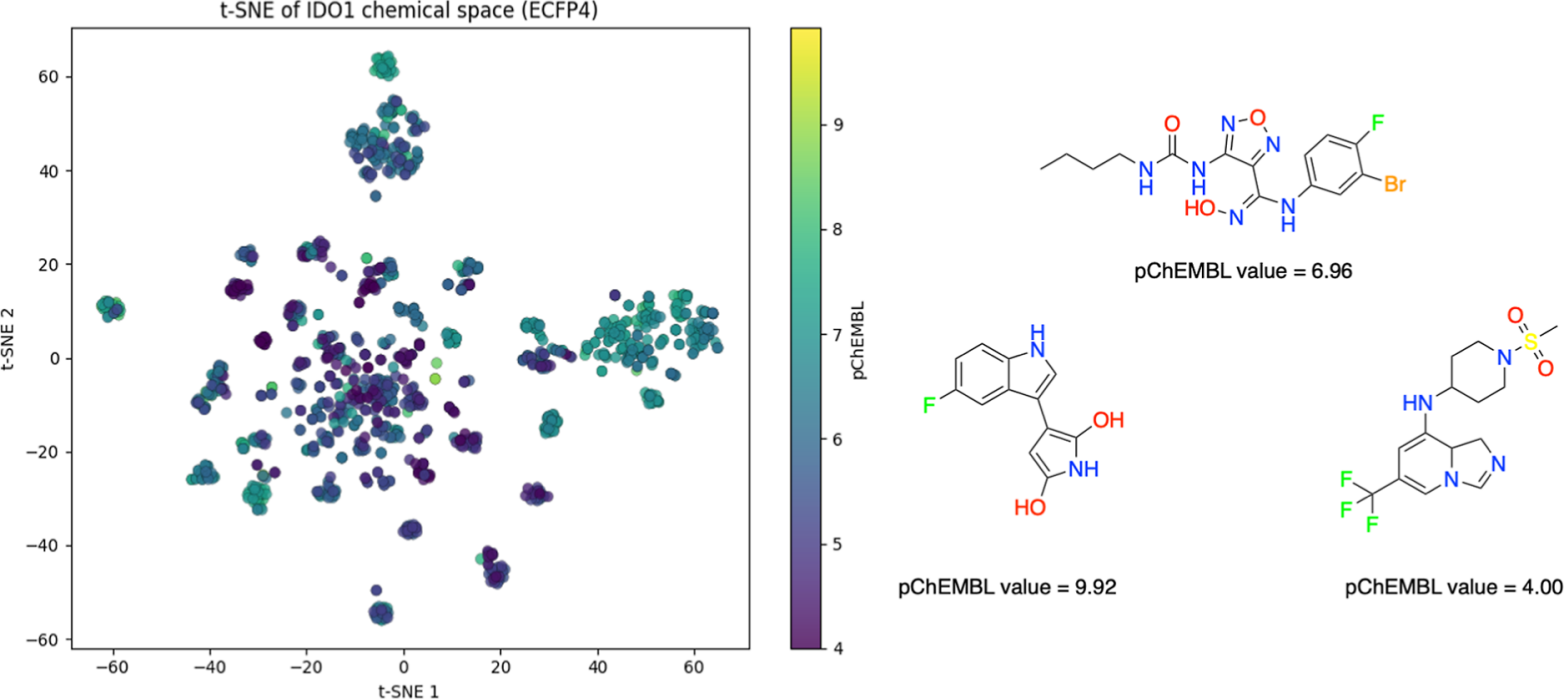
t-SNE analysis of the curated data set, with reported some representative molecules with pChEMBL value.

To systematically assess the influence of molecular representation on predictive robustness, five complementary feature spaces were generated:^18^ Extended-Connectivity FingerPrints with radius 2 (ECFP4), Functional-Class FingerPrint with radius 2 (FCFP4), Molecular ACCess System (MACCS) keys, physicochemical descriptors, and Mordred descriptors.^19^ Circular fingerprints (ECFP4 and FCFP4) encode local atomic environments and are widely recognized as effective representations for ligand-based virtual screening. MACCS keys provide a compact and interpretable substructure-based encoding,^20^ whereas Mordred descriptors capture a broad spectrum of topological, geometric, and electronic properties, enabling global structural characterization.

Continuous descriptor sets were filtered to mitigate redundancy and reduce the risk of overfitting. Near-zero-variance features were removed, and pairwise Pearson correlation filtering (|ρ| > 0.9) was applied. Residual intrafamily correlations among certain descriptor classes were retained because tree-based and kernel-based learners are intrinsically robust to multicollinearity. The correlation structure is shown in Figure 3.

**Figure 3:**
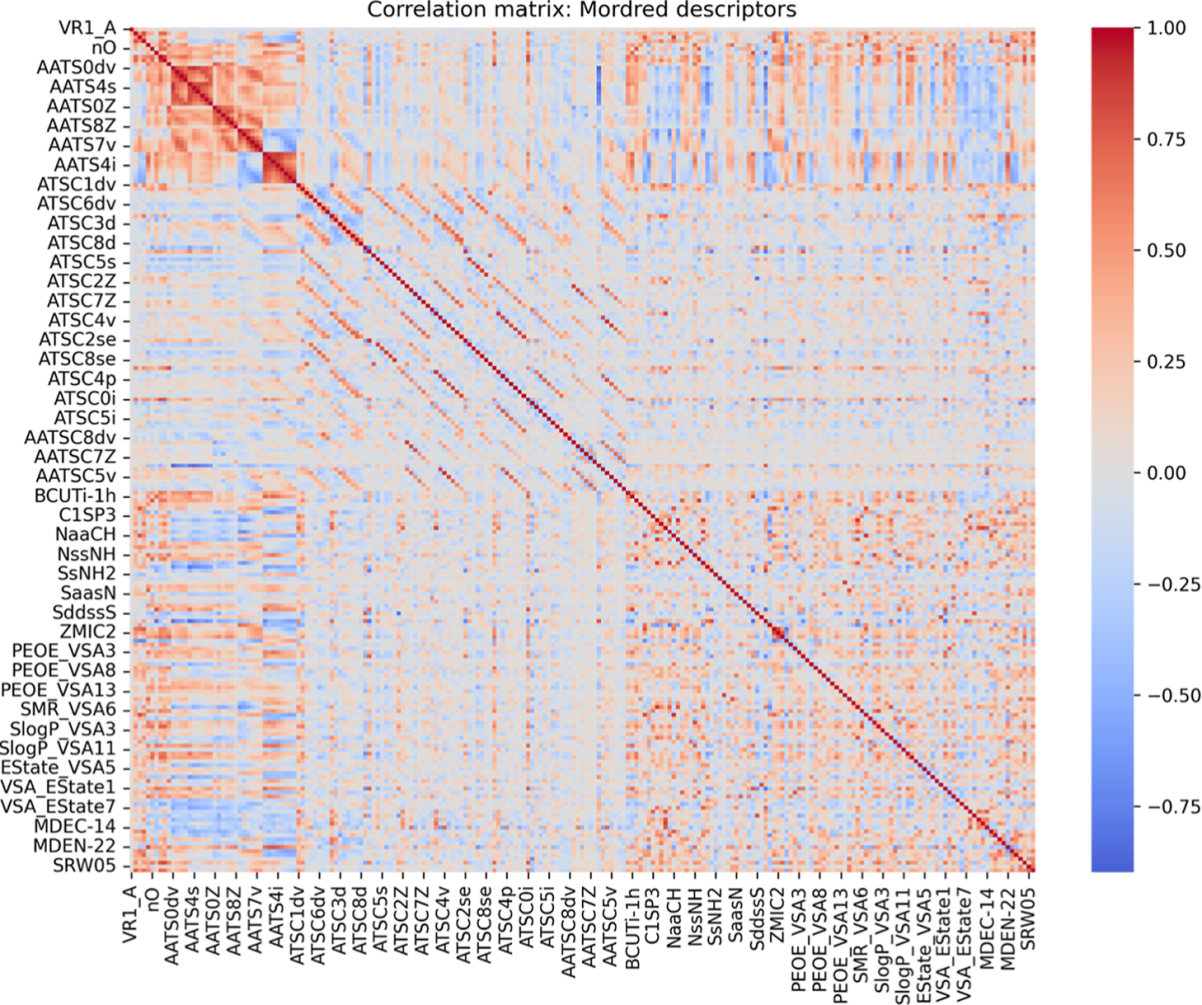
Correlation matrix plots of continuous Mordred descriptors filtered by removing near-constant features and highly correlated variables (|ρ| > 0.9) using a pairwise Pearson correlation criterion.

To obtain a realistic estimate of prospective performance, the data set was divided using a Bemis–Murcko scaffold split. All compounds sharing the same core scaffold were assigned exclusively to either the training or test set, preventing chemical series leakage. Compared with random splitting, scaffold-based evaluation represents a significantly more stringent generalization challenge, as illustrated in Figure 4. This protocol more closely approximates prospective virtual screening, in which predictions are required for unseen chemotypes.

**Figure 4:**
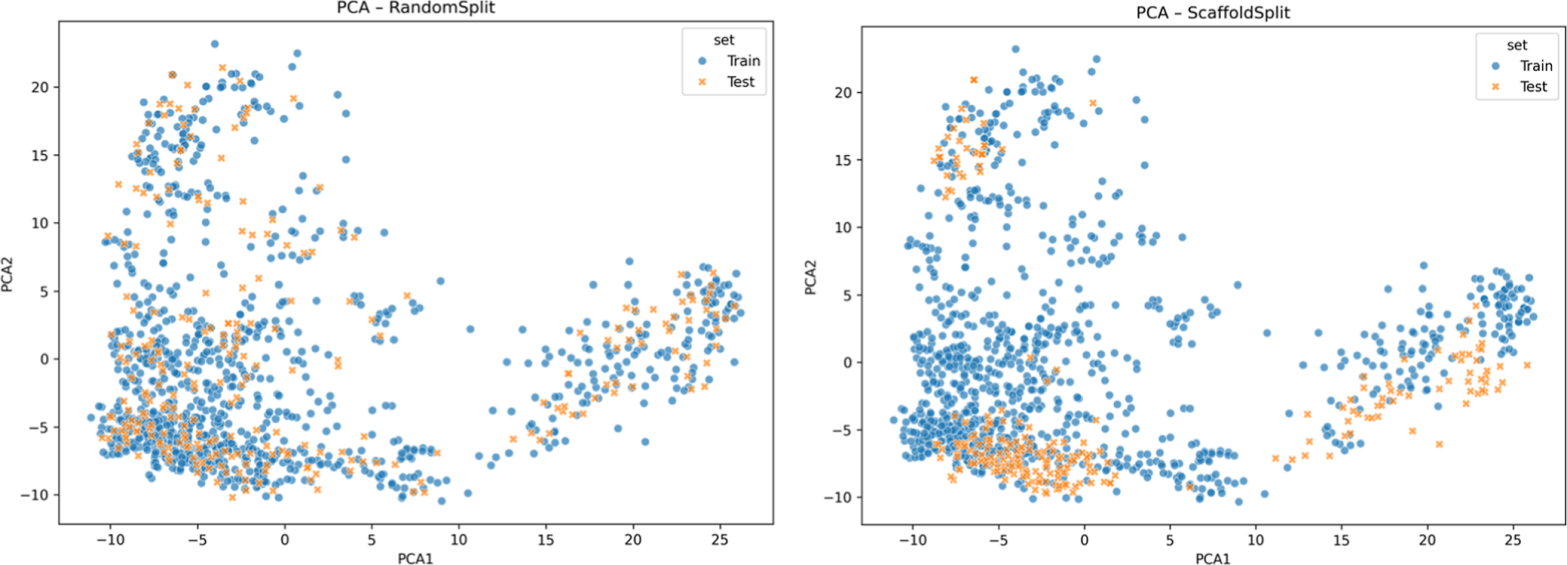
PCA plot of both random and scaffold splitting of the data set.

Three complementary machine learning algorithms were evaluated: Random forest (RF),^21^ extreme Gradient Boosting (XGB),^22^ and support vector machine (SVM).^23^ Hyperparameter optimization was performed using nested cross-validation restricted to the training set to avoid information leakage. Performance metrics were subsequently computed on the external scaffold-based test set to ensure an unbiased generalization assessment (Figure 5).

**Figure 5:**
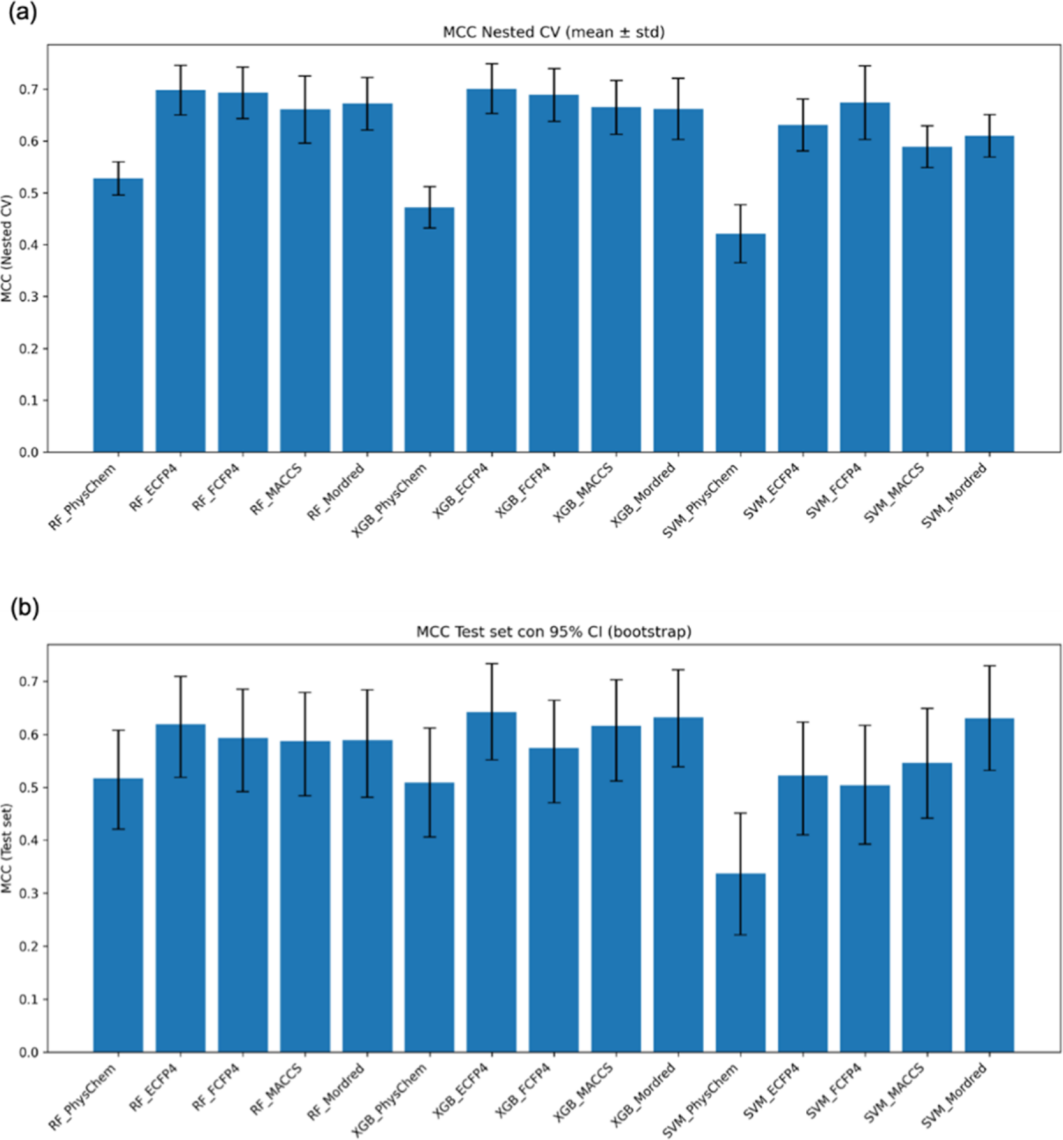
Bar plot of MCC metrics for cross-validation (a) and test (b) for every model–feature block.

Across all algorithms, fingerprint-based models consistently outperformed low-dimensional physicochemical descriptors (Table 1). Models trained exclusively on PhysChem features achieved moderate discrimination (ROC-AUC ≈0.81) but substantially lower MCC values (∼0.50), with pronounced degradation for SVM (MCC = 0.34), indicating limited extrapolative capacity under scaffold splitting. This suggests that global molecular properties alone are insufficient to encode the structural determinants of IDO1 inhibition.

**Table 1:** MCC Results in Both Cross-Validation and Test Sets for Each Couple Model Feature

Algorithm	Feature	MCC CV (mean ± std)	MCC test (95% CI)
	PhysChem	0.53 ± 0.03	0.52 (0.42–0.61)
	ECFP4	0.70 ± 0.04	0.62 (0.52–0.71)
RF	FCFP4	0.69 ± 0.05	0.59 (0.49–0.69)
	MACCS	0.66 ± 0.07	0.59 (0.48–0.68)
	Mordred	0.67 ± 0.05	0.59 (0.48–0.68)
	PhysChem	0.47 ± 0.04	0.51 (0.41–0.61)
	ECFP4	0.70 ± 0.05	0.64 (0.55–0.73)
XGB	FCFP4	0.69 ± 0.05	0.57 (0.47–0.66)
	MACCS	0.67 ± 0.05	0.62 (0.51–0.70)
	Mordred	0.66 ± 0.06	0.63 (0.54–0.72)
	PhysChem	0.42 ± 0.06	0.34 (0.22–0.45)
	ECFP4	0.63 ± 0.05	0.52 (0.41–0.62)
SVM	FCFP4	0.67 ± 0.07	0.50 (0.39–0.62)
	MACCS	0.59 ± 0.04	0.55 (0.44–0.65)
	Mordred	0.61 ± 0.04	0.63 (0.53–0.73)

Among all representations, ECFP4 fingerprints provided the most robust performance, particularly when combined with tree-based learners. The XGB–ECFP4 model achieved an ROC-AUC of 0.884 and an MCC of 0.642 on the external test set, closely followed by RF–ECFP4 (ROC-AUC = 0.890; MCC = 0.619). Narrow confidence intervals indicate stable generalization despite the chemically challenging evaluation regime. These findings reinforce the suitability of high-dimensional circular fingerprints for capturing nonlinear structure–activity relationships.

SVM performance was strongly dependent on the descriptor type. Although SVM models trained on binary fingerprints exhibited acceptable ROC-AUC values, class separation was inferior to that of tree-based methods. In contrast, SVM trained on Mordred descriptors achieved competitive performance (ROC-AUC = 0.879; MCC = 0.630), consistent with the theoretical advantage of kernel methods in dense, continuous feature spaces. This highlights the complementary nature of local substructure encoding (fingerprints) and global molecular descriptors.

FCFP4 and MACCS representations yielded intermediate results. Although discrimination remained high (ROC-AUC typically 0.85–0.89), MCC values were systematically lower than those of ECFP4-based models, suggesting a reduced sensitivity–specificity balance under scaffold-based evaluation. This likely reflects the loss of atom-level specificity in functional-class fingerprints and the limited expressivity of predefined structural keys.

As expected, all models exhibited a measurable decrease in performance when moving from cross-validation to external scaffold-based testing, confirming the increased stringency of the evaluation protocol. Importantly, the top-performing models retained strong predictive power, supporting their suitability for prospective screening.

Probability calibration analysis further supported the superiority of tree-based fingerprint models, which achieved lower Brier scores and better-aligned predicted probabilities.^24^ Well-calibrated outputs are critical in downstream prioritization workflows, in which ranking stability and confidence estimation directly influence decision-making.

### Y-Randomization (QSAR Robustness Test)

Permutation testing (Y-randomization) was performed to rule out chance correlations. Activity labels were randomly shuffled 100 times while preserving the original class distribution, and the full modeling pipeline, including nested cross-validation and hyperparameter optimization, was repeated for each permuted data set. All randomized models yielded ROC–AUC values close to 0.50, and MCC values near zero on the external scaffold-based test set, confirming that the predictive performance of the original models does not arise from descriptor bias or data set imbalance, but reflects genuine structure–activity relationships (Figure S1).

### Weighted Heterogeneous Ensemble

Owing to the complementary strengths and partially nonoverlapping error profiles of individual models, a weighted ensemble was constructed. The ensemble combined RF and XGB models trained on ECFP4 fingerprints with XGB and SVM models trained on Mordred descriptors. Weights were derived from the validation performance to balance discrimination and stability (Table 2).

**Table 2:** Performance Comparison of the Test Set Among Base and Ensemble Models

model	ROC-AUC (95% CI)	accuracy (95% CI)	precision (95% CI)	recall (95% CI)	F1 (95% CI)	MCC (95% CI)
RF_ECFP4	0.89 (0.85–0.93)	0.81 (0.76–0.85)	0.90 (0.85–0.95)	0.79 (0.72–0.85)	0.84 (0.79–0.89)	0.62 (0.52–0.71)
XGB_ECFP4	0.88 (0.84–0.92)	0.82 (0.78–0.87)	0.91 (0.86–0.95)	0.80 (0.74–0.86)	0.85 (0.81–0.89)	0.64 (0.55–0.73)
XGB_mordred	0.88 (0.84–0.92)	0.82 (0.77–0.86)	0.91 (0.86–0.96)	0.78 (0.72–0.85)	0.84 (0.80–0.88)	0.63 (0.54–0.72)
SVM_mordred	0.88 (0.83–0.92)	0.82 (0.78–0.87)	0.89 (0.84–0.93)	0.83 (0.77–0.88)	0.86 (0.81–0.90)	0.63 (0.53–0.73)
ensemble	0.90 (0.85–0.93)	0.82 (0.77–0.86)	0.91 (0.86–0.96)	0.79 (0.72–0.85)	0.85 (0.80–0.89)	0.64 (0.55–0.73)

On the external scaffold-based test set, the ensemble achieved an ROC-AUC of 0.895 (95% CI: 0.852–0.934) and MCC of 0.638 (95% CI: 0.548–0.728), alongside balanced precision (0.913), recall (0.788), and specificity (0.871) (Figure 6). Although the ensemble did not substantially exceed the best-performing single model in absolute MCC, it consistently reduced variance and avoided strong dependence on any single descriptor space or learning bias. This stabilizing effect is particularly valuable under scaffold-based evaluation, where model sensitivity to chemical novelty can lead to unpredictable performance fluctuations. Therefore, the ensemble provides improved robustness and more reliable probability estimates for prospective applications to FDA-approved compound libraries.

**Figure 6:**
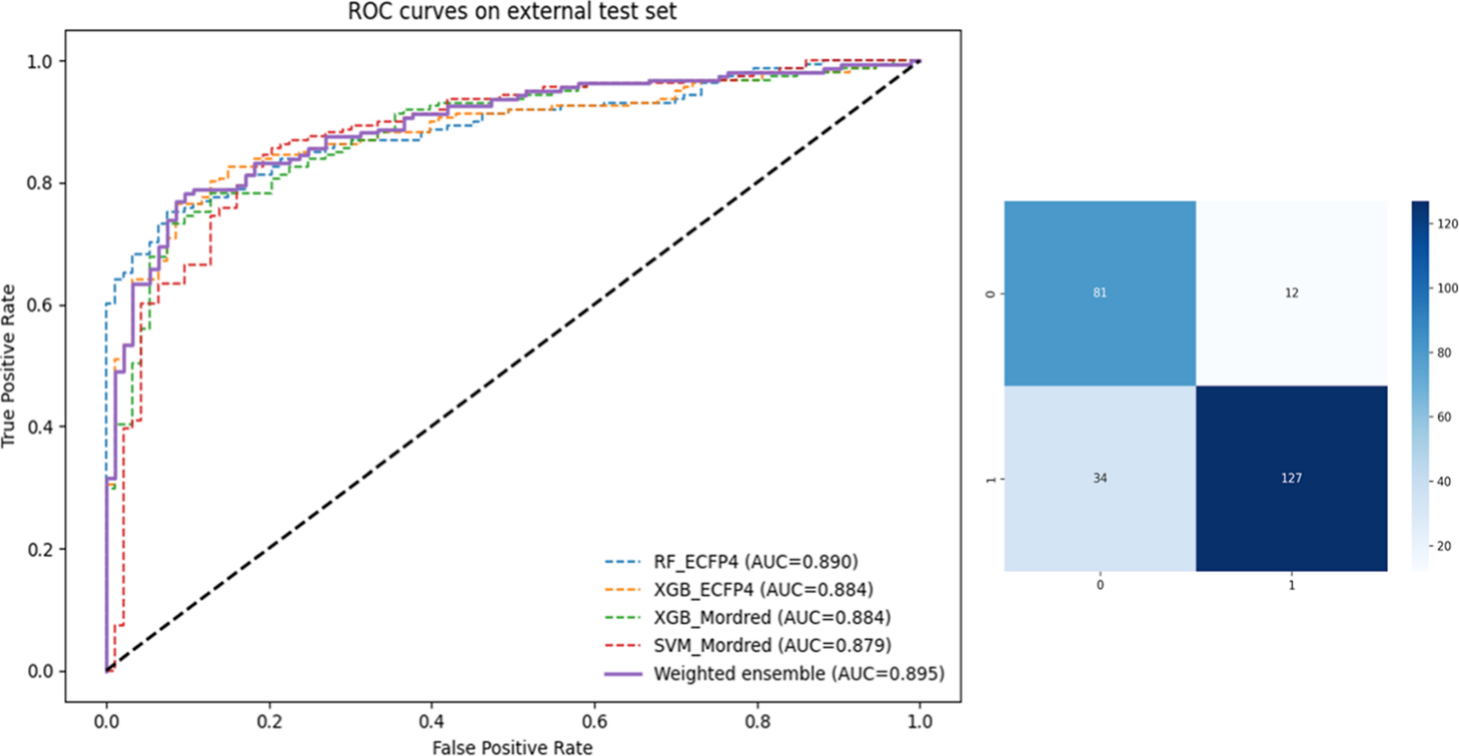
ROC curves of the best-performing base models and the final weighted ensemble on the external test set. For completeness, the ROC curves for all model–feature combinations are reported in Figure S2.

To explicitly quantify the contribution of each base learner, a leave-one-model-out ablation study was performed, in which each model was systematically removed from the ensemble. The results are reported in the Supporting Information (Table S1). Removal of individual models results in only marginal variations in ROC-AUC (0.891–0.897), indicating that no single classifier dominates the ensemble performance.

Slight reductions in performance are observed upon removal of RF- and XGB-based models, suggesting that tree-based learners contribute complementary predictive information across different descriptor representations. In contrast, removal of the SVM-based model leads to a slight increase in MCC (0.672 vs 0.639), indicating that this model introduces partially redundant or mildly conflicting decision boundaries under scaffold-based evaluation. This behavior is consistent with known differences between kernel-based and tree-based learners in high-dimensional chemical descriptor spaces.^25^

Overall, the ablation analysis confirms that the ensemble benefits from the integration of heterogeneous models rather than relying on any single descriptor–algorithm combination. Performance remains stable across perturbations, demonstrating that predictive power arises from complementary and partially overlapping information sources.

Tree-based learners preferentially exploit high-dimensional circular fingerprints, whereas kernel-based methods benefit from rich continuous descriptor spaces. Their integration within a heterogeneous ensemble framework enhances predictive reliability and supports statistically grounded candidate prioritization for downstream structure-based refinement.

### Feature Importance

Feature importance analyses for all models are shown in Figure S3. We focus on the linear SVM model trained on Mordred descriptors (Figure 7), as this representation provides the most interpretable mapping between molecular properties and predicted activity. Descriptor definitions are available in the official Mordred documentation (https://mordred-descriptor.github.io/documentation/master/descriptors.html).

**Figure 7:**
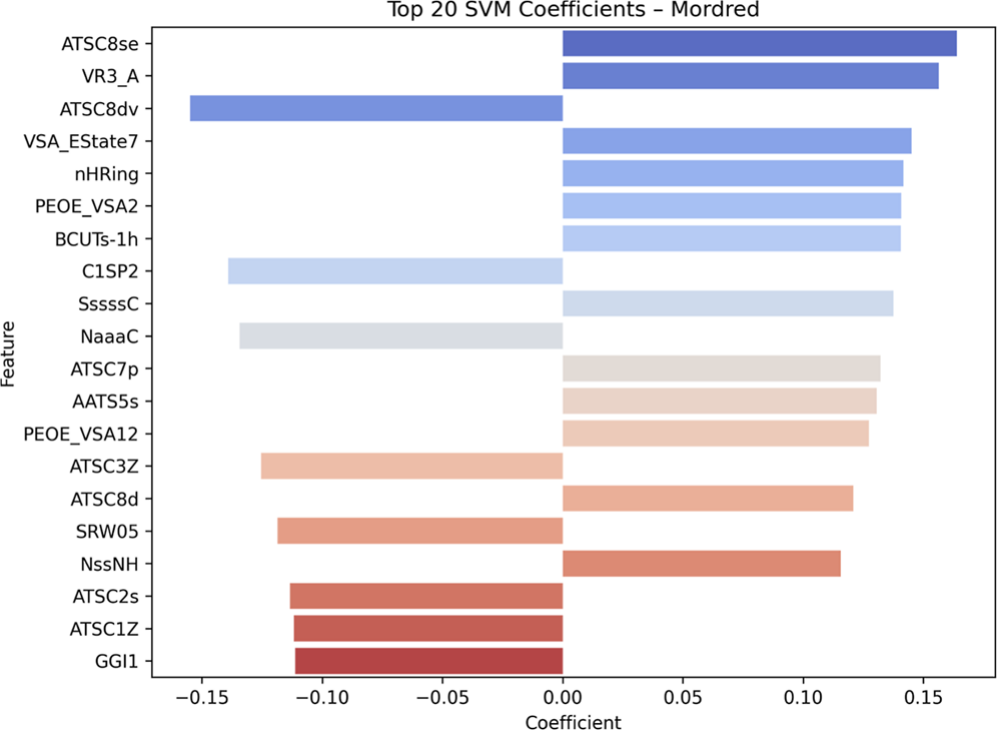
Feature importance analysis of the linear SVM model based on Mordred descriptors. Horizontal bar plot reporting the top 20 Mordred descriptors ranked by the absolute value of their SVM coefficients. Bars extending to the right (positive coefficients) indicate descriptors positively correlated with IDO1 inhibitory activity, whereas bars extending to the left (negative coefficients) indicate descriptors associated with reduced activity. Bar length reflects the magnitude of each descriptor’s contribution to the SVM decision function. Color intensity encodes the sign and magnitude of the coefficients, with blue tones corresponding to positive contributions and red tones to negative contributions.

The linear SVM coefficients reveal a consistent physicochemical signature associated with IDO1 inhibition. Among the positively weighted descriptors, heteroaromatic ring count (nHRing) and multiple electrostatic surface terms (e.g., VSA_EState7, PEOE_VSA2, PEOE_VSA12) are prominent. These descriptors encode the distribution of partial charges across solvent-accessible surface areas and suggest that spatially organized polar surface features and heteroaromatic substructures favorably contribute to classification as active. In addition, several topological autocorrelation and branching descriptors (ATSC8se, ATSC7p, ATSC8d, VR3_A, BCUTs-1h) exhibit positive coefficients, indicating that moderate three-dimensional connectivity and controlled molecular complexity are associated with predicted activity. Collectively, these features point toward compact, heteroatom-rich scaffolds with structured polar surface patterns rather than purely hydrophobic architectures.

Conversely, negatively weighted descriptors included long-range autocorrelation indices and electronic dispersion-related terms (ATSC8dv, ATSC3Z, and ATSC 2s), as well as features associated with specific sp^2^ carbon distributions (C1SP2) and graph invariants (GGI1 and SRW05). These descriptors are often linked to extended planarity, delocalized π-systems, or repetitive topological motifs. Their negative contribution suggests that excessive rigidity or long-range electronic uniformity may reduce compatibility with the IDO1 binding environment under the learned decision boundary.

Notably, similar trends were observed in the XGBoost model trained on the same Mordred descriptor set (Figure S2). In the nonlinear framework, nitrogen count (nN), electrostatic surface descriptors (EState_VSA, PEOE_VSA), and short-range topological autocorrelations emerged as the highest-importance features. The convergence between the linear SVM and nonlinear XGBoost models, despite fundamentally different inductive biases, supports the robustness of the inferred structure–activity patterns. Although individual descriptor coefficients should not be interpreted as causal determinants, the consistency across modeling paradigms indicates that IDO1 inhibition is strongly associated with localized heteroatom content, structured polar surface distribution, and controlled topological complexity.

Overall, the feature importance analysis provides a chemically interpretable rationale for the ensemble predictions and confirms that both local substructural patterns and global physicochemical organization contribute to the classification boundary.

### SHapley Additive exPlanations (SHAP) Analysis

To improve model interpretability and enable both global and sample-level explanations, SHAP analysis was performed on all base learners composing the ensemble. For each model, global feature importance bar plots are reported in the Supporting Information (Figure S4), while the beeswarm plots in Figure 8 summarize the top 20 ranked features according to the mean absolute SHAP value. In these plots, each row represents a feature ordered by global importance, and each dot corresponds to the SHAP value for a single compound. Feature values are color-coded, with high values in red and low values in blue, enabling direct visualization of the directionality of feature contributions toward the predicted class.

**Figure 8:**
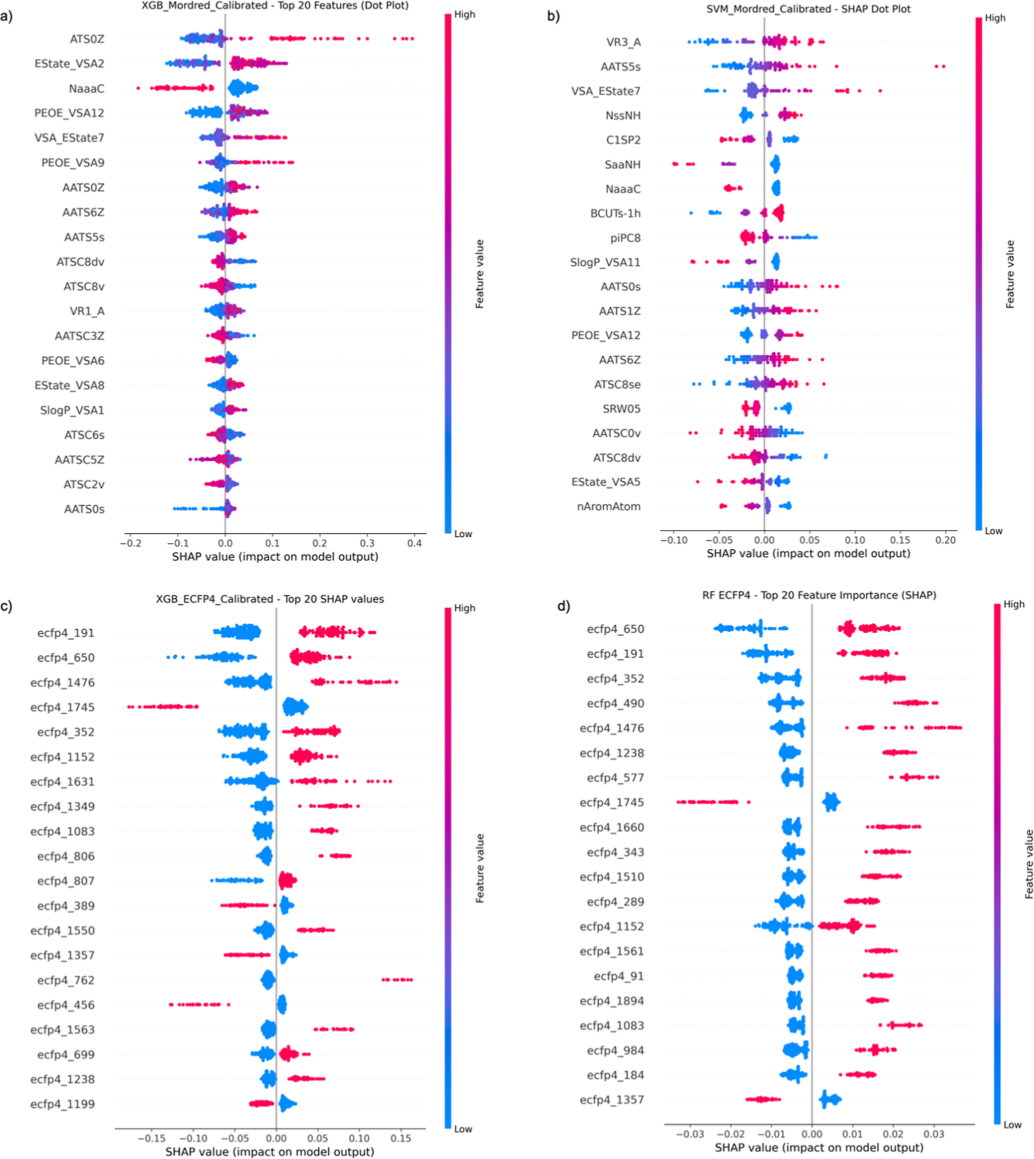
SHAP analysis beeswarm plot of the top 20 features for (a) XGB and (b) SVM with Mordred descriptor; and SHAP analysis beeswarm plot for (c) XGB and (RF) with ECFP4.

For Mordred descriptor-based models, a strong convergence between the linear SVM and the nonlinear XGBoost learners was observed. In both models, the distribution of partial charges across the molecular surface emerged as a primary determinant of activity. Descriptors such as PEOE_VSA12 and VSA_EState7 consistently showed that higher descriptor values shift the prediction toward the active class, supporting the hypothesis that spatially organized polar surface regions are critical for favorable interactions within the IDO1 binding pocket. In contrast, NaaaC (aromatic carbons without attached hydrogens) appeared among the most influential features in both models but with an opposite contribution, where high values consistently produced negative SHAP values. This result indicates that excessive aromatic density or extended π-systems are detrimental to inhibitory potency.

Model-specific patterns provided additional mechanistic insights. In the SVM model, the relevance of VR3_A­(logarithmic Randic index) and AATS 5s confirmed the importance of molecular topology and branching, with higher values favoring activity. Nitrogen-containing descriptors (SaaNH and NssNH) revealed a refined sensitivity to hydrogen-bonding patterns: lower SaaNH values were associated with positive SHAP contributions, suggesting that an optimal spatial arrangement of donor/acceptor groups is more important than their absolute abundance. In the XGBoost model, ATS0Z (atomic number sum) emerged as the most influential descriptor, where high values strongly increased the probability of activity, suggesting the presence of a molecular mass or heavy-atom threshold associated with IDO1 inhibition. This effect was complemented by EState_VSA2, further reinforcing the central role of charge distribution across models.

SHAP analysis of ECFP4 fingerprints also revealed a high degree of consistency between XGBoost and Random Forest models. Both architectures independently identified fragments 191,650, and 352 as key positive contributors to IDO1 inhibition (Figure 9), while fragment 1745 consistently acted as a strong negative determinant, shifting predictions toward inactivity. These findings align with the trends observed from Mordred descriptors, indicating that excessive density of non-hydrogenated aromatic carbons or suboptimal positioning of polar groups in the core scaffold may introduce steric or electrostatic penalties. The strong cross-model agreement supports the robustness of the identified structural motifs as pharmacophoric and antipharmacophoric elements within the explored chemical space.

**Figure 9:**
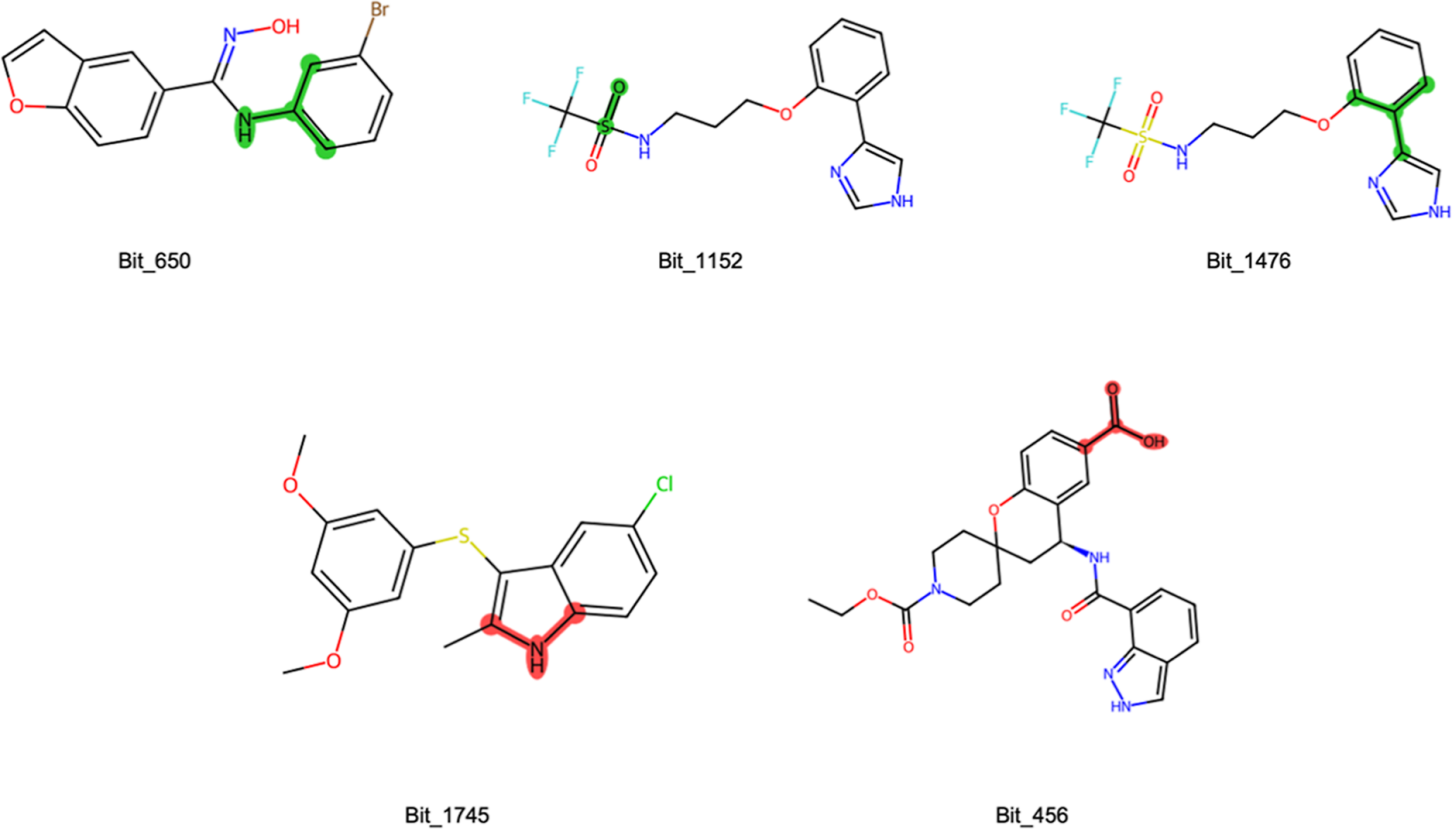
Substructure bit from ECFP4 fingerprint; green fragments represent a positive contribution toward activity, red fragments instead represent negatives contribution.

### Prediction of FDA-Approved Compounds

The FDA-approved compound library was retrieved from Selleck Chemicals (https://www.selleckchem.com/screening/fda-approved-drug-library.html) and curated using the same preprocessing pipeline applied to the training data set to ensure descriptor consistency. Before prediction, the applicability domain (AD) of the ensemble model was assessed to control extrapolation beyond the chemical space represented during training. AD was defined using the Mahalanobis distance in descriptor space, which accounts for the feature covariance structure and provides a multivariate measure of structural similarity to the training distribution. Approximately 70% of the screened compounds were classified within the AD, indicating that the ensemble spans a substantial portion of pharmaceutically relevant chemical space while maintaining conservative extrapolation boundaries.^26^

The predicted probability distributions across ensemble components are shown in Figure 10. High intermodel agreement was observed for the top-ranked compounds, particularly between RF_ECFP4 and XGB_Mordred, which exhibited consistently elevated median confidence values. In contrast, SVM_Mordred displayed a broader probability distribution, effectively acting as a regularizing component within the ensemble by attenuating extreme predictions for borderline cases. This behavior reflects differences in the decision function geometry between tree-based and kernel-based learners and contributes to ensemble stability.

**Figure 10:**
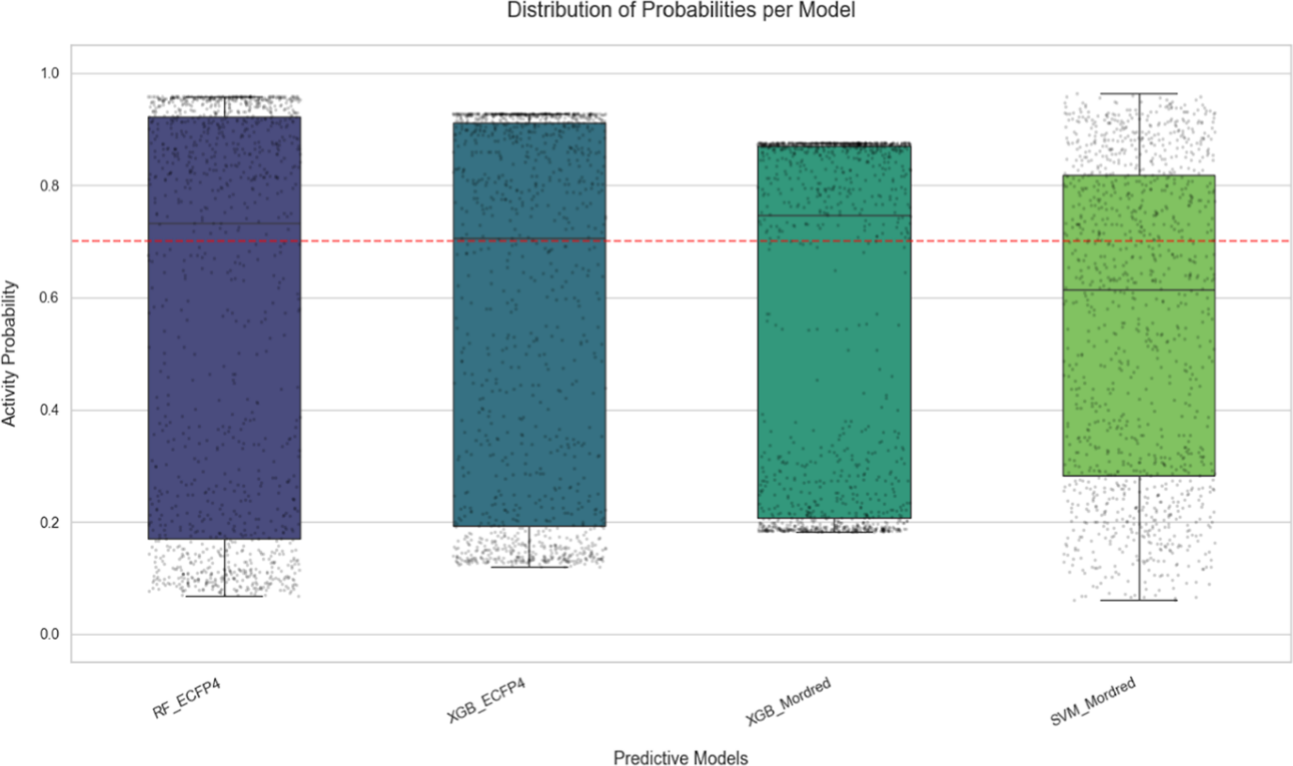
Distribution of predicted probabilities for FDA-approved compounds across ensemble models. The stripplot shows the confidence scores generated by RF_ECFP4, XGB_ECFP4, XGB_Mordred, and SVM_Mordred for each compound. The red dashed line indicates the high-confidence threshold of 0.70 used for candidate selection.

A probability threshold of 0.70 was applied to ensure stringent prioritization. This cutoff was selected to balance precision and recall under the scaffold-based validation regime while minimizing false positives. A distinct cluster of compounds exceeded this threshold consistently across all models, indicating that prioritized candidates are supported by both substructure-driven (fingerprint-based) and physicochemical (descriptor-based) representations. Such multirepresentation agreement reduces susceptibility to model-specific bias and enhances confidence in downstream structure-based refinement.

The application of this filtering criterion yielded 39 compounds within the applicability domain predicted as high-confidence IDO1 inhibitors (Table S2). These candidates were subsequently advanced to ensemble docking for structure-based evaluation, thereby integrating ligand-based prioritization with conformationally aware binding assessment.

### Molecular Docking Analysis

IDO1 exhibits pronounced conformational plasticity within its catalytic cleft, particularly across the A and B subpockets that mediate substrate and inhibitor recognition.^14,15^ Structural rearrangements involve both loop mobility and side-chain reorientation, thereby directly affecting pocket geometry and the electrostatic environment. Multiple crystallographic structures are available in the Protein Data Bank, representing both holo (heme-bound) and apo (heme-free) conformations complexed with chemically diverse ligands.

In the present study, we focused on apo structures to investigate small molecules that are potentially capable of competing with heme binding and stabilizing the heme-free state. Apo-targeting inhibitors (often classified as Type IV) displace the heme cofactor and stabilize the inactive enzyme form, a mechanism associated with prolonged target engagement and enhanced pharmacodynamic persistence. The high prevalence of apo-IDO1 in neoplastic tissues further supports the relevance of this conformational state as a therapeutic target.^27,28^

To explicitly account for receptor conformational heterogeneity, an ensemble docking strategy was adopted.^29^ Three high-resolution apo crystal structures (PDB IDs: 6WJY, 8ABX, and 9S1X; Figure 11) were selected to represent structurally distinct binding-site conformations. Rather than relying on a single rigid receptor model, which is inherently limited in capturing protein flexibility by using multiple receptor structures,^30^ ensemble docking integrates multiple experimentally observed states, thereby mitigating conformation-specific bias and reducing the risk of false negatives.^31^

**Figure 11:**
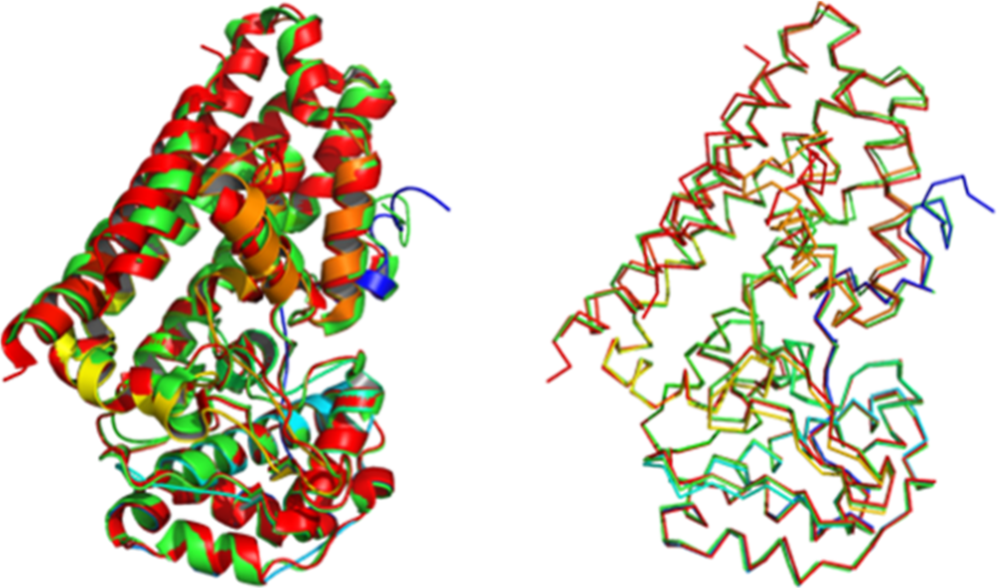
Alignment of selected apo IDO1 crystal structures (6WJY, 8ABX, and 9S1X).

Each crystal structure was retrospectively validated using a curated set of known actives and property-matched decoys. Performance was evaluated using ROC-AUC and early enrichment metrics (EF1%, EF5%, and EF10%). The best-performing structure (6WJY) achieved ROC-AUC values up to 0.97, whereas all selected structures demonstrated acceptable discriminative capacity (AUC >0.70; Table S3), supporting their suitability for prospective screening.

Redocking experiments were conducted to assess pose reproducibility. Root-mean-square deviation (RMSD) values confirmed the accurate recovery of crystallographic binding modes within acceptable thresholds. The GNINA CNN scoring function^32^ provided consistently high pose confidence scores for native ligands, indicating strong agreement between predicted and experimental conformations and supporting the reliability of CNN-based rescoring in this system.

### Ensemble Docking Performance and Consensus Analysis

Single-structure docking revealed heterogeneous predictive behavior across IDO1 conformations, with variability in early enrichment and ranking stability. This variability reflects the intrinsic conformational bias of rigid docking protocols, in which ligands compatible with one structural state may be penalized in another. To improve robustness, multiple consensus-ranking strategies were systematically benchmarked (Figure 12). These included mean Z-score aggregation, rank-based consensus, weighted Z-score schemes incorporating structure-specific performance metrics, and the Best Z-score approach, which retains the most favorable normalized score observed across receptor conformations.

**Figure 12:**
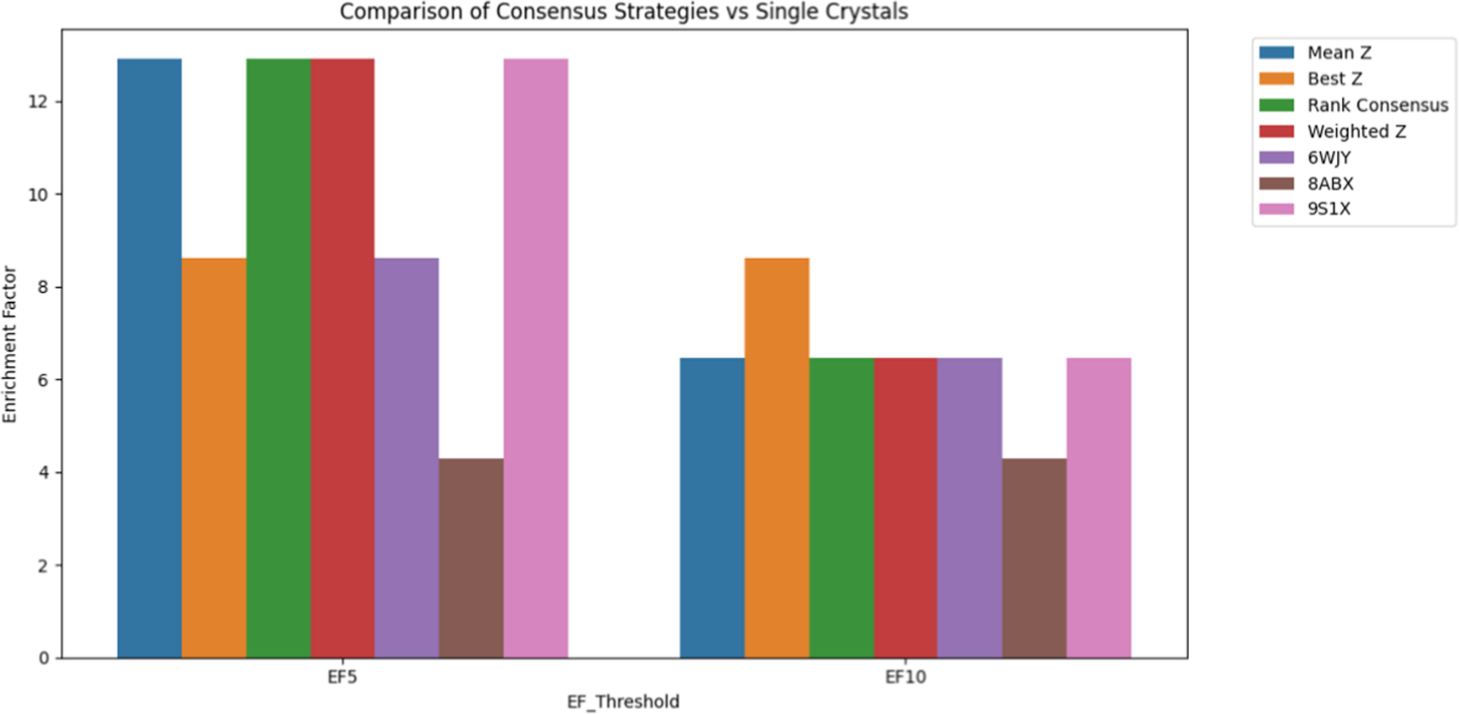
Comparative performance of ensemble docking consensus strategies. The bar plot shows the early enrichment performance (EF5% and EF10%) obtained using different consensus strategies across multiple IDO1 crystal structures.

Although mean- and rank-based consensus strategies improved overall discrimination relative to single-structure docking, they tended to attenuate strong conformation-specific signals. The weighted Z-score method enhanced early enrichment at stringent cutoffs (EF5%) but exhibited reduced EF10%, suggesting partial penalization of ligands with selective conformational affinity. In contrast, the Best Z-score strategy consistently achieved the highest early enrichment at EF10% (EF10 = 8.60) while maintaining competitive ROC–AUC values (Table S4). This indicates that retaining the optimal normalized interaction score across conformations more effectively captures ligands capable of stabilizing at least one binding-competent structural state. Such behavior aligns with a conformational selection paradigm, in which ligands preferentially bind and stabilize specific receptor conformations rather than interacting uniformly across all structural variants.

Based on the retrospective validation results, prospective prioritization was performed using the Best Z-score ranking. To further enhance robustness against structure-specific artifacts, a consistency filter was introduced, retaining compounds ranked within the top 5 or top 10 positions across multiple crystal structures. This combined strategy balances sensitivity to conformation-specific binders with resistance to isolated scoring outliers.

Two distinct categories of prioritized compounds were identified (Table 3 and Figure 13).

**Table 3:** Final Prioritization of Docked Compounds Based on Integrated Consensus Criteria

Rank	Compound	Z-score	CNN affinity (best)	Top 5 consistency	Top 10 consistency
1	Ligand_25	2.72	7.01	2	2
2	Ligand_21	2.40	6.92	1	3
3	Ligand_6	2.16	6.37	3	3
4	Ligand_32	1.85	6.68	1	1
5	Ligand_2	1.75	5.90	1	1
6	Ligand_5	1.73	6.09	3	3
7	Ligand_15	1.43	5.72	1	2
8	Ligand_29	1.36	5.73	2	2
9	Ligand_22	1.26	5.51	1	1
10	Ligand_30	1.13	5.70	0	1

**Figure 13:**
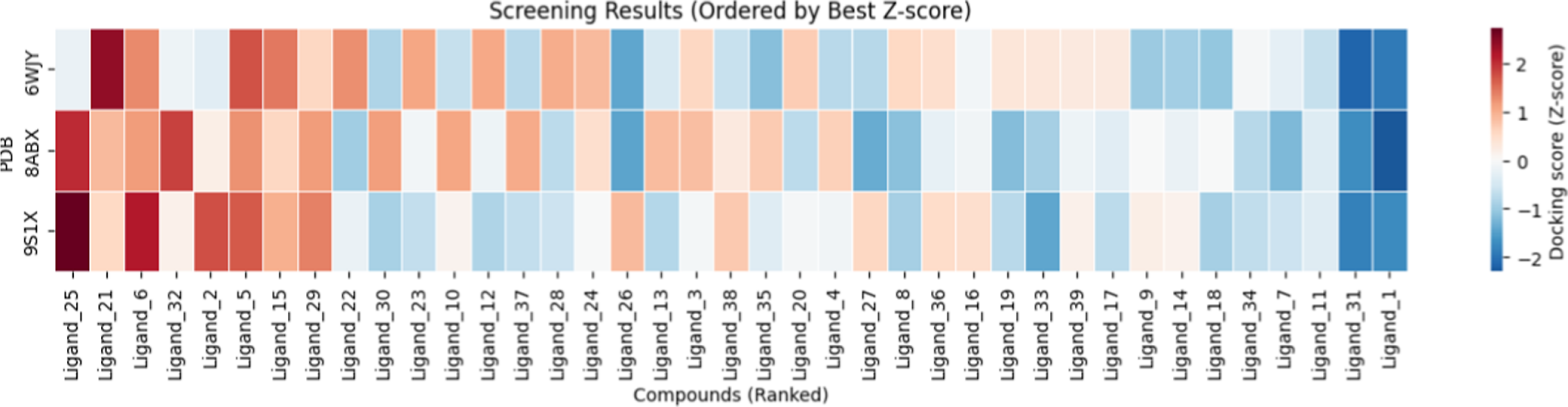
Heatmap of Z-score–normalized docking scores across receptor conformations. The compounds are ranked according to their Best Z-score, which represents the most favorable normalized docking score observed across all receptor conformations.

The first group comprised structurally robust candidates characterized by high Best Z-scores, strong CNN affinity estimates, and consistent top-10 rankings across multiple receptor conformations (e.g., Ligand_25, Ligand_21, Ligand_6). These compounds are insensitive to moderate binding-site variability and are therefore less susceptible to conformational bias, supporting their prioritization for downstream dynamic evaluation. The second group consisted of conformation-selective candidates (e.g., Ligand_32, Ligand_2), which achieved high Best Z-scores and CNN confidence in a single structure but exhibited limited cross-structure consistency. This behavior suggests preferential stabilization of specific IDO1 conformational states. Although more sensitive to receptor geometry, such ligands may exploit transient or functionally relevant conformations that are not uniformly captured across crystallographic snapshots.

To ensure stringent advancement criteria, only compounds with CNN affinity scores >6.3 were selected for molecular dynamics simulations. This cutoff corresponds to predicted low-micromolar to submicromolar binding confidence within the GNINA scoring framework. The integrated selection scheme, which combines normalized docking performance, CNN pose confidence, and cross-structure consistency, provides a conservative and quantitatively benchmarked basis for progression to dynamic refinement.

### Complementarity between Machine Learning and Docking

A moderate divergence was observed between high-confidence machine-learning predictions and docking-derived affinity estimates. This discrepancy reflects the orthogonal nature of the two methodologies. The ML ensemble captures the statistical structure–activity patterns learned from known inhibitors, emphasizing topological and physicochemical similarity. In contrast, docking evaluates three-dimensional steric compatibility and interaction energetics within explicit receptor conformations.

Compounds scoring highly in ML but poorly in docking likely share global pharmacophoric features with known inhibitors, yet fail to achieve favorable geometric accommodation within specific binding-site states. Conversely, compounds with strong docking scores but moderate ML probabilities may represent structurally novel chemotypes capable of favorable binding, despite limited similarity to the training distribution.

The integration of these orthogonal filters, statistical generalization (ML), and physics-based structural evaluation (docking) reduces reliance on any single predictive paradigm and enhances prioritization robustness (Figure 14). Only candidates satisfying both criteria were advanced to molecular dynamics simulations, thereby strengthening confidence in downstream structural refinement.

**Figure 14:**
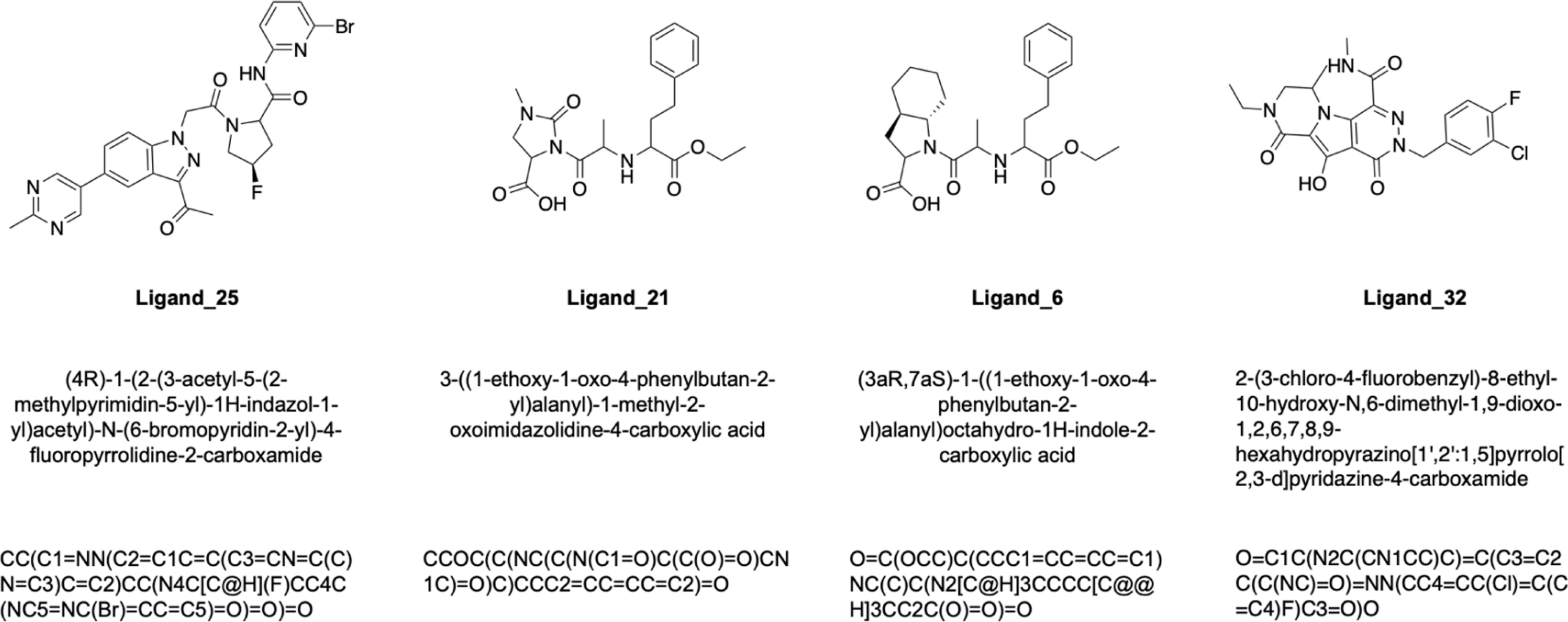
Structural representations and identifiers of the top-ranked compounds selected for MD simulations.

### Baseline Strategies Comparison

To assess the contribution of hierarchical integration, we compared the full workflow with simplified strategies using either the ML ensemble alone or docking on individual crystal structures (6WJY, 8ABX, 9S1X). Ranking solely by ML probabilities (ensemble probability (*P*_ens_) > 0.70) placed only Ligand_6 within the top 10, whereas other compounds selected for MD (Ligand_21, Ligand_25, Ligand_32) fell below this threshold. Single-structure docking improved the identification of some candidates in specific conformations; for example, Ligand_25 ranked within the top five in 8ABX and 9S1X, but other compounds, including Ligand_32, remained outside the top 10 in at least one crystal. Ligand_6 consistently ranked high across all structures, as shown in Figure 15.

**Figure 15:**
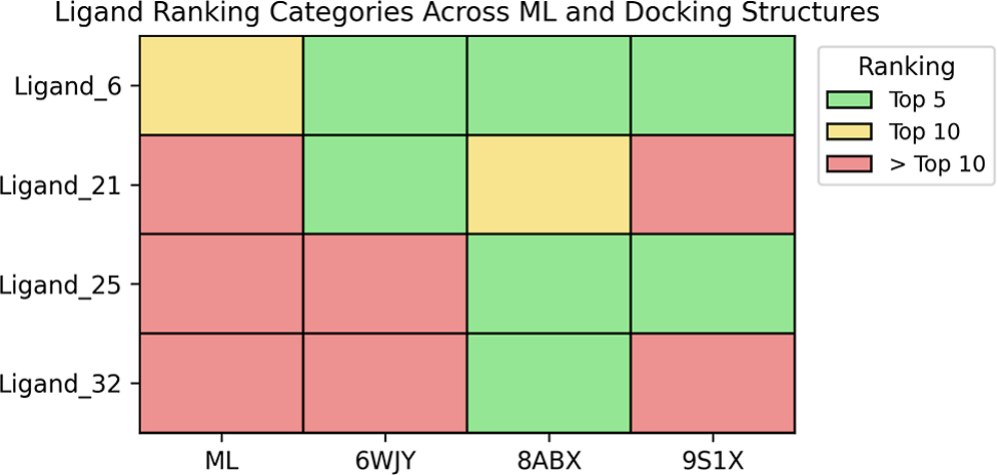
Heatmap representation of the ranking positions obtained using the ML ensemble and single-structure docking on 6WJY, 8ABX, and 9S1X. Each cell reports the ranking category of a ligand within the FDA library: green box indicates placement within the top 5, yellow within the top 10, and red beyond the top 10.

In contrast, the integrated workflow, which combines ML predictions with multistructure docking and hierarchical cross-structure filtering, successfully prioritized all compounds selected for the MD. These results indicate that neither ML alone nor docking on individual crystal structures captures the full set of top candidates, and that hierarchical integration provides a more robust and reproducible selection framework for downstream dynamic evaluation.

#### Homology Modeling and Molecular Dynamics Simulations

The available apo IDO1 crystal structures contain unresolved segments, particularly within flexible loop regions. Because long-time scale molecular dynamics (MD) simulations require a structurally complete model to avoid artificial flexibility at truncated termini or missing regions, a homology modeling procedure was performed to reconstruct the full-length protein. The inclusion of these segments was considered necessary to reduce boundary artifacts and to better capture long-range conformational coupling that may indirectly influence active-site geometry during extended simulations.

The resulting model (Figure 16) preserved the experimentally resolved structural core while incorporating missing regions based on template-guided modeling. Structural alignment with the reference crystal (6WJY) confirmed the preservation of the catalytic cleft architecture, ensuring that the docking-derived binding poses remained structurally consistent with the experimental framework.

**Figure 16:**
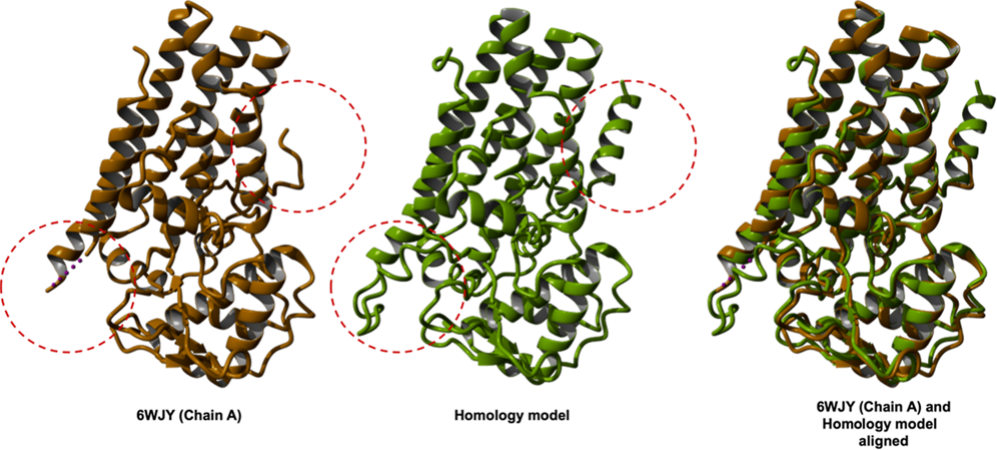
Homology model of full-length IDO1 (green) compared to the 6WJY template (brown). The regions derived by homology modeling that were missing in the experimental protein model are highlighted by a red dotted circle.

Using the reconstructed full-length model, 300 ns all-atom MD simulations were performed for the four prioritized ligands. For each compound, the top-ranked docking pose from the best-performing crystal structure was used as the initial configuration (Ligand_6 and Ligand_25 from 9S1X; Ligand_21 from 6WJY; Ligand_32 from 8ABX).

System stability was monitored through potential energy and backbone RMSD relative to the minimized starting structure (Figure S5). In all simulations, the potential energy profiles fluctuated around stable mean values without systematic drift, indicating equilibration of the systems. RMSD trajectories displayed an initial relaxation phase, followed by plateau behavior for Ligand_25 and Ligand_32, suggesting convergence toward stable conformational ensembles. In contrast, RMSD fluctuations were broader for Ligand_6 and Ligand_21, indicative of less stable binding or increased conformational rearrangement within the binding pocket.

Although RMSD alone does not fully characterize binding stability, the combined absence of energy drift and sustained RMSD plateaus for Ligand_25 and Ligand_32 supports the maintenance of their docked orientations over most of the simulation time. Therefore, these two systems were prioritized for further structural refinement and analysis.

### Molecular Mechanics/Poisson–Boltzmann Surface Area Binding Free Energy Estimation

To complement the RMSD-based stability assessment with an energetic metric, end point Molecular Mechanics/Poisson–Boltzmann Surface Area (MM/PBSA) binding free energy calculations were performed on snapshots extracted every 10 ns from the final 200 ns of each trajectory (Table S5). Because Ligand_6 and Ligand_21 failed to reach a stable RMSD plateau, they were excluded from this thermodynamic analysis to prevent the derivation of unreliable metrics. Relative binding free energies (Δ*G*_bind_) for the stable systems were computed using the Poisson–Boltzmann implicit solvent model implemented in YASARA. Ligand_25 and Ligand_32 displayed highly favorable average binding energies, in agreement with their superior structural stability and redocking performance. Although MM/PBSA values should be interpreted comparatively rather than as absolute thermodynamic quantities, these energetic profiles strongly support the hierarchical prioritization obtained from the ensemble docking.

### RMSF and Contact Persistence Analysis

Root-mean-square fluctuation (RMSF) analysis was conducted to evaluate residue-level flexibility across the simulation trajectories (Figure S6, left panel). Binding-site residues interacting with Ligand_25 and Ligand_32 exhibited reduced fluctuation amplitudes relative to the other complexes, particularly in the 260–265 loop region and around His346. In addition, hydrogen-bond and hydrophobic contact occupancies were monitored throughout the trajectories (Figure S6, right panel). Persistent interactions (>50% occupancy) were observed for key residues identified in the docking analysis, supporting the structural relevance of the predicted binding modes under dynamic conditions.

### Redocking Analysis

To evaluate the structural impact of MD refinement on the binding-site geometry, an ensemble-based post-MD redocking protocol was implemented to reassess predicted affinities across multiple dynamically relaxed states.^33^

Instead of relying on a single final snapshot, the MD trajectories of the prioritized systems were clustered from their respective equilibration points onward (after 60 ns for Ligand_25 and 120 ns for Ligand_32) based on structural similarity. This clustering procedure yielded a representative set of dynamically equilibrated receptor conformations for each complex: 5 clusters for Ligand_25 and 8 clusters for Ligand_32. For each extracted snapshot, the ligand was removed, and the minimized protein conformation was used for redocking following the same parameters applied during the initial virtual screening phase. Redocking performance was subsequently evaluated by calculating both the CNN_VS affinity score and the RMSD of the predicted pose relative to its corresponding MD conformation. Both compounds exhibited high average CNN_VS scores and remarkably low positional deviations (Table 4). The subangstrom RMSD values indicate that the redocked poses are highly conserved and display excellent structural compatibility with the dynamically relaxed receptor environment. This improvement in structural complementarity following MD relaxation strongly supports their prioritization as robust candidates.

**Table 4:** Average Redocking Performance Calculated Across all Cluster Representatives Extracted from the MD Trajectories^a^ ^,^ ^b^

Compound	Number of clusters	CNN_VS	RMSD (Å)
Ligand_25	5	8.88 ± 0.23	0.28 ± 0.16
Ligand_32	8	7.17 ± 0.28	0.63 ± 0.08

a Data are expressed as Mean ± Standard Deviation for both the predicted affinity (CNN_VS) and the positional deviation (RMSD) of the redocked poses relative to their corresponding MD conformations.

b The new grid parameters are shown in Table S6, and the complete redocking data are presented in Tables S7 and S8.

Based on the integrated evidence from ensemble docking, MD stability, and redocking refinement, danicopan (Ligand_25), approved for the treatment of adults with paroxysmal nocturnal hemoglobinuria (PNH),^34^ and MK-2048 (Ligand_32), a second-generation integrase inhibitor active against HIV,^35^ emerged as the most robust candidates within the screened FDA-approved library.

Rather than asserting definitive inhibitory activity, these compounds were proposed as prioritized candidates for experimental validation within an apo-IDO1 targeting framework.

### Ligand–Protein Interactions Analysis

A detailed interaction analysis of the redocked poses for Ligand_25 and Ligand_32 revealed both shared anchoring features and distinct binding orientations (Figure 17). Ligand_25 predominantly occupied Pocket A and established a structured hydrogen-bond network involving Ala264, His346, and Cys129. The interaction with His346 is particularly notable, as it combines hydrogen bonding with π–π stacking, potentially reinforcing positional stability near the heme-binding region. The hydrophobic substituent of Ligand_25 is embedded within a lipophilic subpocket formed by Leu339, Leu342, Leu384, and Val269, enabling multiple alkyl and π–alkyl contacts that contribute to hydrophobic complementarity.

**Figure 17:**
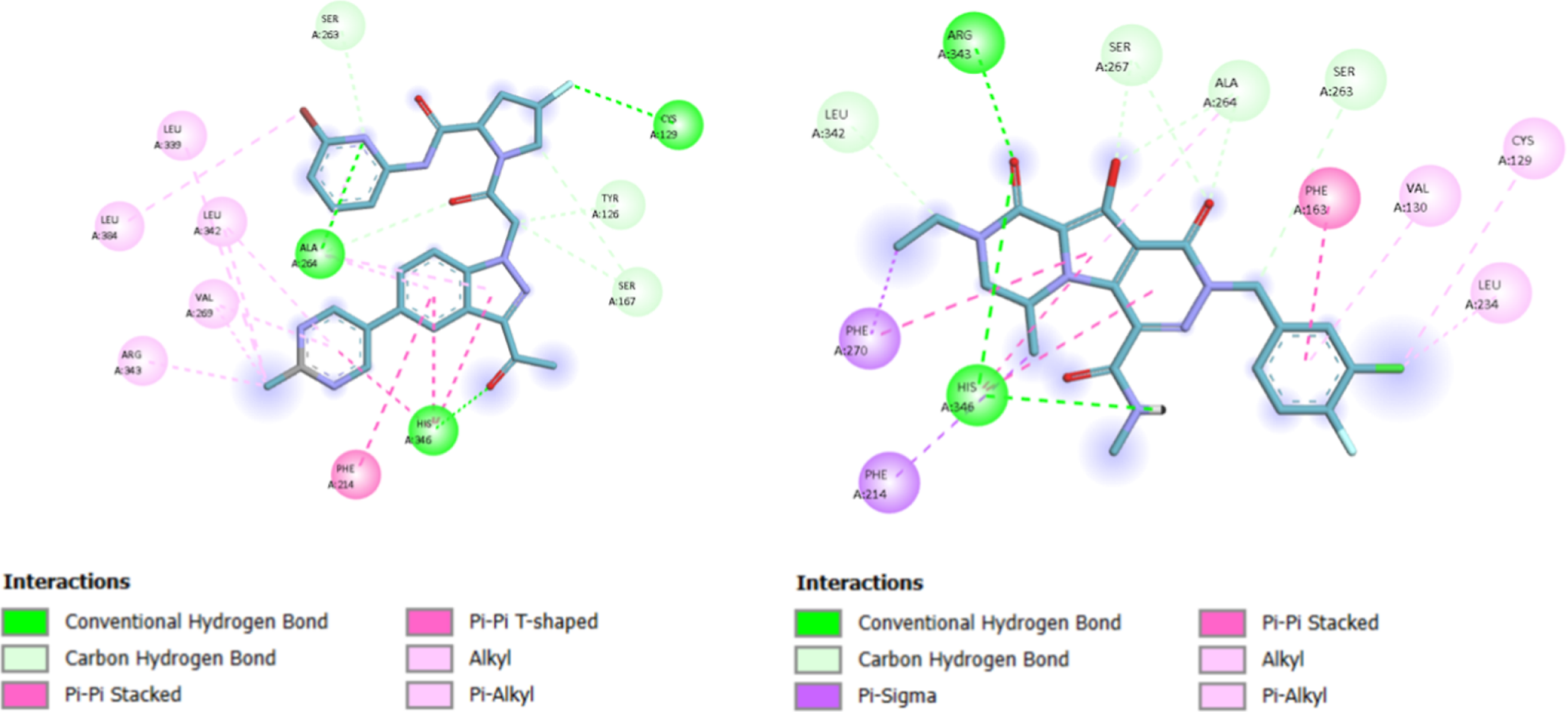
Ligand interaction maps for Ligand_25 (left) and Ligand_32 (right).

Ligand_32 adopts a partially shifted orientation, engaging Arg343 and His346 via hydrogen bonds while forming π–σ interactions with Phe270 and Phe214. These aromatic contacts may enhance stabilization within the flexible pocket environment. Compared to Ligand_25, Ligand_32 extends toward distal residues such as Leu234 and Val130, suggesting an alternative exploitation of the subpocket topology.

Both ligands interact with the Ser263–Ala264 region, a structural element implicated in ligand accommodation within apo conformations. The recurrent involvement of His346 in both complexes reinforces its role as a central recognition residue within the IDO1 active site.

## Material and Methods

### Data Curation and Machine Learning Models Development

#### Data Retrieval and Curation

Molecular data targeting the enzyme indoleamine 2,3-dioxygenase 1 (IDO1), UniProt accession P14902, were systematically retrieved from the ChEMBL database (ChEMBL ID: CHEMBL4685) on December 15, 2025. The obtained data set underwent a rigorous curation process following best practices in cheminformatics. Only ChEMBL records that reported a half-maximal inhibitory concentration (IC_50_) value and an associated pChEMBL value were retained, with the pChEMBL value serving as a standardized, comparable measure of compound potency. Compound selection was restricted to assays performed in a single protein format. To maintain a focus on the drug-like chemical space, compounds violating more than two Lipinski’s Rule of Five (RO5) rules were also excluded. An RDKit-based Python script was subsequently utilized to clean the data by identifying and removing duplicates based on their canonical SMILES strings, along with any compounds possessing missing or ambiguous structural information. Molecules were standardized according to the recommended ChEMBL preprocessing workflow.^36^ The pChEMBL value distribution was analyzed, and activity outliers were statistically identified using a modified Z-score based on the median absolute deviation (MAD). A stringent threshold of |*M*| > 3.5 was applied, and any molecules identified as outliers were removed from the final data set. Given that the objective of the classification model is to identify potent compounds with submicromolar activity, capable of competing with the heme cofactor,^37^ the curated compounds were categorized into two distinct activity classes based on their standardized potency: compounds with a pChEMBL value ≥6 were labeled as Active (1), while those with a pChEMBL value < 6 were designated as Inactive (0).^38^ Activity distribution is shown in Figure S7. This process yielded a final data set consisting of 1277 entries, which were specifically divided into 735 active and 542 inactive compounds.

### Chemical Space Analysis

An initial chemical space analysis was conducted on the final data set to visualize the diversity and distribution of the compounds. This was achieved by employing t-distributed Stochastic Neighbor Embedding (t-SNE) on the Extended Connectivity Fingerprints (ECFP4). The resulting t-SNE plot was visualized by mapping the continuous pChEMBL values to a color gradient.

### Molecular Descriptor Calculation

The physical chemical descriptors, ECFP4, FCFP4, MACCs Key, and Mordered descriptors were calculated using RDKit (v. 2025.9.3), and Mordred (v. 1.2.0) packages.^19^ Low-variance and highly correlated features (|*r*| > 0.9) were removed only from continuous descriptors (physicochemical and Mordred). Binary fingerprints were filtered exclusively by removing constant bits. Physicochemical descriptors were standardized after filtering.

The data set was split into training (80%) and external test (20%) sets using a scaffold-based splitting procedure. All model selection and tuning steps were performed using the training set alone.

### Machine Learning Algorithm Training and Validation

To classify molecules based on IDO1 activity, supervised machine learning (ML) models were trained using multiple molecular representations, including ECFP4, FCFP4, MACCS fingerprints, Mordred descriptors, and physicochemical properties. The algorithms employed were Random Forest (RF), XGBoost (XGB), and Support Vector Machine (SVM). For each couple (algorithm-descriptor block), a model was trained and validated using a nested cross-validation (CV) strategy (5-fold outer, 3-fold inner) to ensure unbiased performance estimation and robust grid search hyperparameter optimization. Within each outer training fold, hyperparameters were optimized, selecting configurations that maximized the ROC-AUC metric (Table S9).

After nested cross-validation, the most frequently selected hyperparameter configuration was used to retrain the model on the full training set, and the final performance was evaluated on the external test set. Probability calibration was performed using Platt scaling (sigmoid method) to ensure reliable output scores. The statistical significance of the test set metrics was evaluated through 1000 bootstrap iterations to calculate 95% confidence intervals. The reliability of the model output probabilities was assessed using the Brier score and calibration plots.

Performance was evaluated using ROC–AUC, MCC, F1-score, precision, recall (sensitivity), specificity, and accuracy. These metrics were computed from the confusion matrix defined by true positives (TP), true negatives (TN), false positives (FP), and false negatives (FN), where TP denotes active compounds correctly predicted as active, TN denotes inactive compounds correctly predicted as inactive, FP denotes inactive compounds incorrectly predicted as active, and FN denotes active compounds incorrectly predicted as inactive.

The metrics are defined as follows

$$accuracy=\frac{TP+TN}{TP+TN+FP+FN}$$

$$precision=\frac{TP}{TP+FP}$$

$$recall\left(TPR\right)=\frac{TP}{TP+FN}$$

$$specificity=\frac{TN}{TN+FP}$$

$$F1-score=2\times \frac{\left(precision\times recall\right)}{\left(precision+recall\right)}$$

$$MCC=\frac{TP\times TN-FP\times FN}{\sqrt{\left(TP+FP\right)\left(TP+FN\right)\left(TN+FP\right)\left(TN+FN\right)}}$$

$$TPR=\frac{TP}{TP+FN}$$

$$FPR=\frac{FP}{FP+TN}$$

The ROC curve was constructed by plotting TPR as a function of FPR across varying classification thresholds, and the area under the curve (ROC–AUC) was computed as

$$ROC-AUC=\int_{0}^{1}TPR\left(FPR\right)d\left(FPR\right)$$

To improve robustness and reduce dependency on a single algorithm or feature block, a Weighted Ensemble Classifier was implemented. Base learners were selected based on stable cross-validated MCC and consistent external test performance, retaining only feature block–model combinations showing robust generalization. In this approach, the calibrated probabilistic predictions of individual models are combined using weights derived from their performance (MCC) in cross-validation. Weights were normalized so that their sum equals one, producing aggregated predictions that are more stable.

$$w_{i}=\frac{{MCC}_{i}}{\sum MCC}$$

The final *P*_ens_ for each compound was calculated as the weighted average of the probabilities generated by the constituent models, as defined in the following equation

$$P_{ens}\left(y=1|x\right)=\sum_{i=1}^{n}w_{i}p_{i}\left(y=1|x\right)$$

where:

- *p*_*i*_ denotes the activity probability predicted by each model.
- *w*_*i*_ denotes the normalized weight assigned to each model, reflecting its individual performance and discriminative power established during the validation phase.

This ensemble approach was adopted to mitigate the biases inherent in single-algorithm predictions and enhance the overall robustness of the VS pipeline.

### Applicability Domain (AD)

The applicability domain was defined using the Mahalanobis distance computed in a PCA-reduced descriptor space (20 components), with the threshold set at the 95th percentile of the training distribution.

The 95th percentile was chosen as a compromise between model coverage and extrapolation risk, in line with best practice.^17^

### Molecular Docking Analysis

#### Data Retrieval

Protein models (PDB IDs: 6WJY, 8ABX, and 9S1X) were retrieved from the protein data bank (https://www.rcsb.org/, accessed on 18/12/2025). Poor active ligand SMILES were retrieved from the CHEMBL Web site (https://www.ebi.ac.uk/chembl/, accessed on 20/12/2025) using CHEMBL4685 as the target ID.

### Protein Preparation

The protein was prepared in YASARA software (v. 25.1.13, YASARA Biosciences GmbH, Vienna, Austria)^39^ by adding hydrogens using YASARA’s “Clean → All” option, which adds missing atoms, side chains, and hydrogen atoms while ensuring correct protonation states at physiological pH. Finally, the cocrystallized ligand and water molecules were removed.

### Ligands Preparation

The ligands were prepared from SMILES codes using Open Babel (v. 3.1.1),^40^ where 3D structures were generated and minimized at physiological pH (7.4). Then, 3D structures were optimized at the GFN2 semiempirical level using the xTB (extended tight-binding, v. 6.7.1) package.^41^ Geometry optimization was performed using an analytical linearized Poisson–Boltzmann (ALPB) model for water, with charge states specified for each molecule based on physiological pH.

### Molecular Docking Analysis and Compound Ranking

Molecular docking calculations were performed using GNINA (version 1.3), a deep-learning-augmented docking engine that integrates convolutional neural network (CNN)–based scoring functions.^32,42^ Docking was conducted in the rescored mode, generating 15 ligand pose rotations per compound, with poses ranked according to the CNN score. Protein structures were provided in the PDB format, whereas ligands were supplied as SDF files. Docking grids were defined using the AutoDock Tools (version 1.5.7), centering the simulation box on the cocrystallized ligand of each IDO1 structure. Grid dimensions were deliberately chosen to be sufficiently large to avoid imposing artificial constraints on ligand binding and to allow full exploration of the active site. Detailed grid parameters for each crystal structure are reported in Table S10.

To account for receptor conformational variability, docking was performed against multiple IDO1 crystal structures. CNN docking scores obtained from different receptor conformations are not directly comparable, as each structure produces a distinct score distribution due to differences in binding-site geometry and scoring bias. CNN scores were standardized independently for each crystal structure using Z-score normalization

$$Z_{ij}=\frac{{CNN}_{ij}-\mu_{i}}{\sigma_{i}}$$

where CNN_*ij*_ represents the CNN docking score of compound *j* in crystal structure *i*, and μ_*i*_ and σ_*i*_ correspond to the mean and standard deviation of CNN scores calculated across the screened compound library for that crystal structure. Higher Z-scores indicate more favorable predicted binding interactions.

Compounds were ranked using a consensus strategy based on the best Z-score achieved across all receptor conformations

$$Z_{j}^{best}=\max\left(Z_{ij}\right)$$

complemented by analysis of the CNN score and ranking consistency across the three crystals.

Consistency was defined as the frequency with which a compound was ranked within the top 10 across individual docking runs. Compounds exhibiting both high *Z*_*j*_^best^-score and favorable CNN scores were prioritized. In particular, molecules with a CNN affinity greater than 6.3 were selected for subsequent molecular dynamics simulations, as this threshold has been associated with a higher likelihood of true binding activity in GNINA-based docking workflows.^7^

### RMSD Calculation

RMSD calculations were performed using the obrms command in Open Babel (v. 3.1.1). The cocrystallized ligand and docking output poses are provided in the PDB format.

### ROC Curves and EFs

The ROC curves and corresponding AUC values were computed using the roc_curve­() and auc­() functions implemented in the sklearn.metrics module (scikit-learn v. 1.3.0). For each protein structure, a retrospective validation set comprising 86 compounds was assembled, including five known apo-IDO1 active ligands,^43^ and 81 compounds with low or negligible activity (experimental affinity >1 μM, retrieved from the ChEMBL database), which were treated as negatives for ranking-based evaluation. Binary class labels were assigned accordingly (active = positive, low or negligible active = negative), and ROC–AUC, true positive rate (TPR), and false positive rate (FPR) were calculated from the continuous docking scores generated by each method. The ROC curve was obtained by varying the decision threshold over the full range of scores, and the AUC was computed using trapezoidal numerical integration. ROC plots were generated using matplotlib (v. 3.7.1), reporting TPR as a function of FPR for each docking protocol, with corresponding AUC values indicated in the legend. A diagonal reference line representing random classification (AUC = 0.5) was included to facilitate visual comparison of model performance.

EFs were computed at 1%, 5%, and 10% of the overall data set. For each predictive approach, the compounds were ranked in descending order according to their scores. The number of active molecules within the top-ranked fraction was divided by the number of actives expected from a random selection of equal size, following eq (3)

$$EF=\frac{\left(N\text{\_}active\text{\_}top/N\text{\_}top\right)}{\left(N\text{\_}active\text{\_}totals/N\text{\_}totals\right)}$$

where N_active_top denotes the number of active compounds within the selected subset, N_top corresponds to the size of that subset, N_active_totals represents the total number of active compounds in the data set, and N_totals indicates the overall number of compounds in the data set.

### Crystal Structure Homology Model

Regions that were not resolved in the crystallographic structure were reconstructed through homology modeling based on sequence alignment and structurally compatible fragments, generating a hybrid full-length IDO1 model. Model building and refinement were performed using the integrated *pm_build.mcr* macro implemented in YASARA under default parameters.

The full-length human IDO1 amino acid sequence was retrieved from UniProt (accession code: P14902; https://www.uniprot.org/) and used as an input for homology modeling in the FASTA format. The experimentally resolved crystal structure of PDB 6WJY (chain A) was selected as the primary structural template to preserve the native architecture of the ligand-binding region.

### Molecular Dynamics Simulations

Molecular dynamics (MD) simulations were performed using the YASARA software package. A cubic simulation box was defined, extending 5 Å beyond all protein atoms, and periodic boundary conditions were applied along all spatial directions. MD simulations were executed using the integrated *md_runfast* macro. The simulations were conducted at physiological pH in an aqueous solution containing 0.9% NaCl at a temperature of 298 K and a water density of 0.997 g/mL. An 8 Å cutoff was applied for van der Waals interactions, whereas electrostatic interactions were treated without a distance cutoff by employing the long-range Coulomb algorithm. The simulations were performed for 300 ns using the ff14SB force field. Upon completion of the MD simulations, the *md_analyze* macro was employed to assess RMSD, RMSF, and internal energy fluctuations over time, as well as to perform trajectory clustering based on a 2 Å heavy-atom RMSD threshold. Subsequently, thermodynamic MM/PBSA evaluations were carried out via the *md_analyzebindenergy* macro. The implementation of these built-in YASARA tools maintains standardized parametrization, ensuring a highly reproducible computational protocol.

## Conclusion

We developed and systematically evaluated a hierarchical computational workflow integrating scaffold-aware machine learning–based QSAR, ensemble docking with CNN rescoring, consensus ranking strategies, and long-time scale molecular dynamics simulations for the prioritization of IDO1 inhibitors. Combined ligand-based and structure-based strategies were designed to explicitly mitigate common methodological limitations in virtual screening, including training-set bias, chemical series leakage, and docking sensitivity to receptor conformational variability.

QSAR prefiltering enabled the efficient reduction of chemical space by selecting compounds consistent with the structural and physicochemical determinants of known IDO1 inhibitors while operating within a defined applicability domain, modeling docking across multiple crystal structures, coupled with CNN-based scoring and systematic benchmarking of consensus approaches, demonstrated that Z-score–based aggregation improved early enrichment and reduced structure-specific artifacts inherent to rigid-receptor models. Molecular dynamics simulations extended beyond static docking predictions and confirmed the persistence of key ligand–target interactions over extended simulation time scales, supporting the structural stability of the predicted binding modes.

Among the screened FDA-approved compounds, danicopan (Ligand_25) and MK-2048 (Ligand_32) emerged as top-ranked candidates, exhibiting stable RMSD trajectories, sustained interaction networks within the catalytic pocket, and consistently favorable CNN-based affinity estimates across docking and redocking analyses. Although experimental validation is required to confirm inhibitory activity, these findings illustrate the ability of the proposed workflow to prioritize chemically plausible candidates in a statistically controlled and reproducible manner.

Overall, this study demonstrates that the hierarchical integration of scaffold-aware machine learning, ensemble structure-based evaluation, and dynamic refinement provides a robust and transferable framework for inhibitor prioritization, particularly for conformationally flexible targets. The modular architecture of the workflow supports adaptation to other systems and enables transparent, quantitatively benchmarked decision-making in computational drug discovery and drug repurposing campaigns.

## Supplementary Material



## Data Availability

Data and Software Availability: all data and scripts required to reproduce the machine learning training, ensemble prediction, Y-randomization experiments, and docking analyses are available at: https://github.com/rocco-b/IDO1-inhibitors-ML-and-docking-data. The repository includes curated data sets, model training and prediction pipelines, a reproducible Jupyter notebook, and a Streamlit application for external predictions.

## Notes

Additional methodological details, data set description, Y-randomization results, ablation study, SHAP analysis, pChEMBL distribution, docking validation metrics, redocking parameters, MM/PBSA calculations, and supplementary figures and tables (PDF)

## References

[1] Cherkasov A., Muratov E. N., Fourches D., Varnek A., Baskin I. I., Cronin M., Dearden J., Gramatica P., Martin Y. C., Todeschini R., Consonni V., Kuz’min V. E., Cramer R., Benigni R., Yang C., Rathman J., Terfloth L., Gasteiger J., Richard A., Tropsha A. (2014). QSAR Modeling:
Where Have You Been? Where Are You Going To?. J. Med. Chem..

[2] Li H., Peng J., Sidorov P., Leung Y., Leung K.-S., Wong M.-H., Lu G., Ballester P. J. (2019). Classical
Scoring Functions for Docking Are Unable to Exploit Large Volumes
of Structural and Interaction Data. Bioinformatics.

[3] Catacutan D. B., Alexander J., Arnold A., Stokes J. M. (2024). Machine Learning
in Preclinical Drug Discovery. Nat. Chem. Biol..

[4] Fuadah Y. N., Pramudito M. A., Firdaus L., Vanheusden F. J., Lim K. M. (2024). QSAR Classification
Modeling Using Machine Learning
with a Consensus-Based Approach for Multivariate Chemical Hazard End
Points. ACS Omega.

[5] Lombardo L., Battisti V., Langer T., Gitto R., De Luca L. (2025). Prediction
of Activity and Selectivity Profiles of Sigma Receptor Ligands Using
Machine Learning Approaches. J. Chem. Inf. Model..

[6] Sivula T., Yetukuri L., Kalliokoski T., Käsnänen H., Poso A., Pöhner I. (2023). Machine Learning-Boosted
Docking
Enables the Efficient Structure-Based Virtual Screening of Giga-Scale
Enumerated Chemical Libraries. J. Chem. Inf.
Model..

[7] Buccheri R., Rescifina A. (2025). High-Throughput, High-Quality: Benchmarking GNINA and
AutoDock Vina for Precision Virtual Screening Workflow. Molecules.

[8] Huang J., Fan X. (2011). Why QSAR Fails: An
Empirical Evaluation Using Conventional Computational
Approach. Mol. Pharmaceutics.

[9] Godin-Ethier J., Hanafi L.-A., Piccirillo C. A., Lapointe R. (2011). Indoleamine 2,3-Dioxygenase
Expression in Human Cancers: Clinical and Immunologic Perspectives. Clin. Cancer Res..

[10] Uyttenhove C., Pilotte L., Théate I., Stroobant V., Colau D., Parmentier N., Boon T., Van Den
Eynde B. J. (2003). Evidence for a Tumoral Immune Resistance Mechanism
Based on Tryptophan Degradation by Indoleamine 2,3-Dioxygenase. Nat. Med..

[11] Long G. V., Dummer R., Hamid O., Gajewski T. F., Caglevic C., Dalle S., Arance A., Carlino M. S., Grob J.-J., Kim T. M., Demidov L., Robert C., Larkin J., Anderson J. R., Maleski J., Jones M., Diede S. J., Mitchell T. C. (2019). Epacadostat plus Pembrolizumab versus Placebo plus
Pembrolizumab in Patients with Unresectable or Metastatic Melanoma
(ECHO-301/KEYNOTE-252): A Phase 3, Randomised, Double-Blind Study. Lancet Oncol..

[12] Feng X., Liao D., Liu D., Ping A., Li Z., Bian J. (2020). Development of Indoleamine 2,3-Dioxygenase 1 Inhibitors
for Cancer
Therapy and Beyond: A Recent Perspective. J.
Med. Chem..

[13] Nelp M. T., Kates P. A., Hunt J. T., Newitt J. A., Balog A., Maley D., Zhu X., Abell L., Allentoff A., Borzilleri R., Lewis H. A., Lin Z., Seitz S. P., Yan C., Groves J. T. (2018). Immune-Modulating Enzyme Indoleamine 2,3-Dioxygenase
Is Effectively Inhibited by Targeting Its Apo-Form. Proc. Natl. Acad. Sci. U. S. A..

[14] Röhrig U. F., Majjigapu S. R., Vogel P., Zoete V., Michielin O. (2015). Challenges
in the Discovery of Indoleamine 2,3-Dioxygenase 1 (IDO1) Inhibitors. J. Med. Chem..

[15] Röhrig U. F., Michielin O., Zoete V. (2021). Structure and Plasticity
of Indoleamine
2,3-Dioxygenase 1 (IDO1). J. Med. Chem..

[16] Pushpakom S., Iorio F., Eyers P. A., Escott K. J., Hopper S., Wells A., Doig A., Guilliams T., Latimer J., McNamee C., Norris A., Sanseau P., Cavalla D., Pirmohamed M. (2019). Drug Repurposing:
Progress, Challenges
and Recommendations. Nat. Rev. Drug Discovery.

[17] Tropsha A. (2010). Best Practices
for QSAR Model Development, Validation, and Exploitation. Mol. Inf..

[18] Chuang K. V., Gunsalus L. M., Keiser M. J. (2020). Learning Molecular Representations
for Medicinal Chemistry: Miniperspective. J.
Med. Chem..

[19] Moriwaki H., Tian Y.-S., Kawashita N., Takagi T. M. (2018). A Molecular Descriptor
Calculator. J. Cheminf..

[20] Chen X., Reynolds C. H. (2002). Performance of Similarity Measures
in 2D Fragment-Based
Similarity Searching: Comparison of Structural Descriptors and Similarity
Coefficients. J. Chem. Inf. Comput. Sci..

[21] Breiman L. (2001). Random Forests. Mach. Learn..

[22] Chen T., Guestrin C. (2016). XGBoost: A Scalable
Tree Boosting System. arxiv.

[23] Awad, M. ; Khanna, R. Support Vector Machines for Classification. In Efficient Learning Machines; Apress: Berkeley, CA, 2015; pp 39–66. 10.1007/978-1-4302-5990-9_3.

[24] Lix L. M. (2010). Brier Score
Summarizes Model Calibration and Discrimination - Reply. J. Clin. Epidemiol..

[25] Rodríguez-Pérez R., Bajorath J. (2022). Evolution of Support Vector Machine and Regression
Modeling in Chemoinformatics and Drug Discovery. J. Comput.-Aided Mol. Des..

[26] Gadaleta D., Mangiatordi G. F., Catto M., Carotti A., Nicolotti O. (2016). Applicability
Domain for QSAR Models: Where Theory Meets Reality. Int. J. Quant. Struct.-Prop. Relat..

[27] Cen L., Wu Y., He M., Huang J., Ren W., Liu B., Meng L., Huang L., Gu H., Xu Y., Zhu Q., Zou Y. (2025). Discovery and Optimization of Novel Apo-IDO1 Inhibitors
by a Pharmacophore-Based Structural Simplification Strategy. J. Med. Chem..

[28] Yin Y., He M., Yue J., Li Y., Peng J., Luo X., Wang Z., He X., Liang S., Liu Z., Wang Y. (2025). Identification of a Novel Core Structure of Apo-Ido1 Inhibitors Through
Virtual Screening and Preliminary Hit Optimization. J. Chem. Inf. Model..

[29] Amaro R. E., Baudry J., Chodera J., Demir O. ¨., McCammon J. A., Miao Y., Smith J. C. (2018). Ensemble Docking
in Drug Discovery. Biophys. J..

[30] Evangelista
Falcon W., Ellingson S. R., Smith J. C., Baudry J. (2019). Ensemble Docking
in Drug Discovery: How Many Protein Configurations from Molecular
Dynamics Simulations Are Needed To Reproduce Known Ligand Binding?. J. Phys. Chem. B.

[31] Huang S., Zou X. (2007). Ensemble Docking of Multiple Protein
Structures: Considering Protein
Structural Variations in Molecular Docking. Proteins.

[32] McNutt A. T., Francoeur P., Aggarwal R., Masuda T., Meli R., Ragoza M., Sunseri J., Koes D. R. (2021). GNINA 1.0: Molecular
Docking with Deep Learning. J. Cheminf..

[33] Lexa K. W., Carlson H. A. (2012). Protein Flexibility
in Docking and Surface Mapping. Q. Rev. Biophys..

[34] Kang C. (2024). Danicopan:
First Approval. Drugs.

[35] Van
Wesenbeeck L., Rondelez E., Feyaerts M., Verheyen A., Van Der Borght K., Smits V., Cleybergh C., De Wolf H., Van Baelen K., Stuyver L. J. (2011). Cross-Resistance
Profile Determination of Two Second-Generation HIV-1 Integrase Inhibitors
Using a Panel of Recombinant Viruses Derived from Raltegravir-Treated
Clinical Isolates. Antimicrob. Agents Chemother..

[36] Bento A. P., Hersey A., Félix E., Landrum G., Gaulton A., Atkinson F., Bellis L. J., De Veij M., Leach A. R. (2020). An Open
Source Chemical Structure Curation Pipeline Using RDKit. J. Cheminf..

[37] Röhrig U. F., Reynaud A., Majjigapu S. R., Vogel P., Pojer F., Zoete V. (2019). Inhibition Mechanisms
of Indoleamine 2,3-Dioxygenase 1 (IDO1). J.
Med. Chem..

[38] Mysinger M. M., Carchia M., Irwin J. J., Shoichet B. K. (2012). Directory of Useful
Decoys, Enhanced (DUD-E): Better Ligands and Decoys for Better Benchmarking. J. Med. Chem..

[39] Krieger E., Vriend G. (2014). YASARA View—Molecular Graphics for All Devices—from
Smartphones to Workstations. Bioinformatics.

[40] O’Boyle N. M., Banck M., James C. A., Morley C., Vandermeersch T., Hutchison G. R. (2011). Open Babel:
An Open Chemical Toolbox. J. Cheminf..

[41] Bannwarth C., Caldeweyher E., Ehlert S., Hansen A., Pracht P., Seibert J., Spicher S., Grimme S. (2021). Extended tight-binding
Quantum Chemistry Methods. WIREs Comput. Mol.
Sci..

[42] McNutt A. T., Li Y., Meli R., Aggarwal R., Koes D. R. (2025). GNINA 1.3: The next
Increment in Molecular Docking with Deep Learning. J. Cheminf..

[43] Sun L. (2020). Advances in
the Discovery and Development of Selective Heme-Displacing IDO1 Inhibitors. Expert Opin. Drug Discovery.

