# Supplementary material for: A Reproducible Hierarchical Virtual Screening Framework Integrating Scaffold-Aware Machine Learning, Ensemble Docking, and Molecular Dynamics: Application to IDO1
Source: J Chem Inf Model. 2026 May 29;66(12):7003–20. doi: 10.1021/acs.jcim.6c00967 (PMC13292220; doi:10.1021/acs.jcim.6c00967)
Supplement: Supplementary file 1 [file ci6c00967_si_001.pdf]

Supporting information

A Reproducible Hierarchical Virtual Screening Framework Integrating Scaffold-Aware Machine Learning, Ensemble Docking, and Molecular Dynamics: Application to IDO1

Elisabetta Grazia Tomarchio[a,b], Rocco Buccheri[a], and Antonio Rescifina,[a,*]

[a]*Department of Drug and Health Sciences, University of Catania, Viale A. Doria 6, 95125, Catania, Italy*
[b]*Department of Biomedical and Biotechnological Sciences, University of Catania, Via Santa Sofia 97, 95123 Catania, Italy*

Table of content

- **Figure S1.** Distribution of the mean performance values obtained from Y-permutation tests for each QSAR model (Panels A–C).
- **Figure S2.** Receiver operating characteristic (ROC) curves for each QSAR model.
- **Figure S3.** Feature importance analysis of machine learning models (Panels A–C).
- **Figure S4.** SHAP bar plot analysis for each algorithm–descriptor combination used in the Ensemble model.
- **Figure S5.** Left: total potential energy of the system as a function of time (300 ns) for the four ligand–protein complexes. Right: solute RMSD relative to the reference structure over 300 ns. Panels: (a) Ligand_6, (b) Ligand_21, (c) Ligand_25, and (d) Ligand _32.
- **Figure S6.** Left: root-mean-square fluctuation (RMSF) per protein residue, calculated from the average RMSF of the atoms constituting each residue. Right: number of protein–ligand contacts per residue as a function of simulation time. Panels: (a) Ligand_6, (b) Ligand_21, (c) Ligand_25, and (d) Ligand_32.
- **Figure S7.** pChEMBL pCheMBL activity distribution.
- **Table S1.** Leave-one-model-out ablation results.
- **Table S2.** Canonical SMILES codes and ensemble probability values of FDA-approved molecules predicted as active compound with high confidence (probability >0.7).
- **Table S3.** Docking validation results, including RMSD, CNN score, and predicted binding affinity from the redocking procedure, together with ROC–AUC and enrichment factor (EF) values derived from active/negative discrimination analysis.
- **Table S4.** Comparative analysis of ROC–AUC and enrichment factor (EF) values for active compound recognition obtained from individual crystal structures and ensemble consensus ranking approaches.
- **Table S5.** Relative MM/PBSA binding free energies (ΔG_{bind}) of the studied protein-ligand complexes. Values are expressed in kcal/mol and represent the mean ± standard deviation (std) calculated from snapshots extracted every 10 ns over the final 200 ns of the molecular dynamics' trajectories. YASARA returns binding free energy values with a positive sign, so a more positive value is considered thermodynamically more stable.
- **Table S6.** Grid parameters adapted for redocking performed on each cluster. Npts values for each coordinate were ever equal to 30 and spacing was ever equal to 1. The table shows the selected snapshot for each cluster and its corresponding time of simulation
- **Table S7.** Redocking performance calculated across all cluster representatives extracted from the MD trajectories. Data show the predicted affinity (CNN_VS) and the positional deviation (RMSD) of the redocked poses relative to their corresponding MD conformations of Ligand_25.

- **Table S8.** Redocking performance calculated across all cluster representatives extracted from the MD trajectories. Data show the predicted affinity (CNN_VS) and the positional deviation (RMSD) of the redocked poses relative to their corresponding MD conformations of Ligand_32.
- **Table S9.** Selected best hyperparameters for each algorithm-feature block. For the SVM algorithm (not shown in the table), the only parameter present, svm_C, was equal to 0.01 for all features.
- **Table S10.** Grid box parameters used for each crystal structure in the docking simulations. The spacing was set to 1 in all cases.

Panel A – Random Forest Algorithm

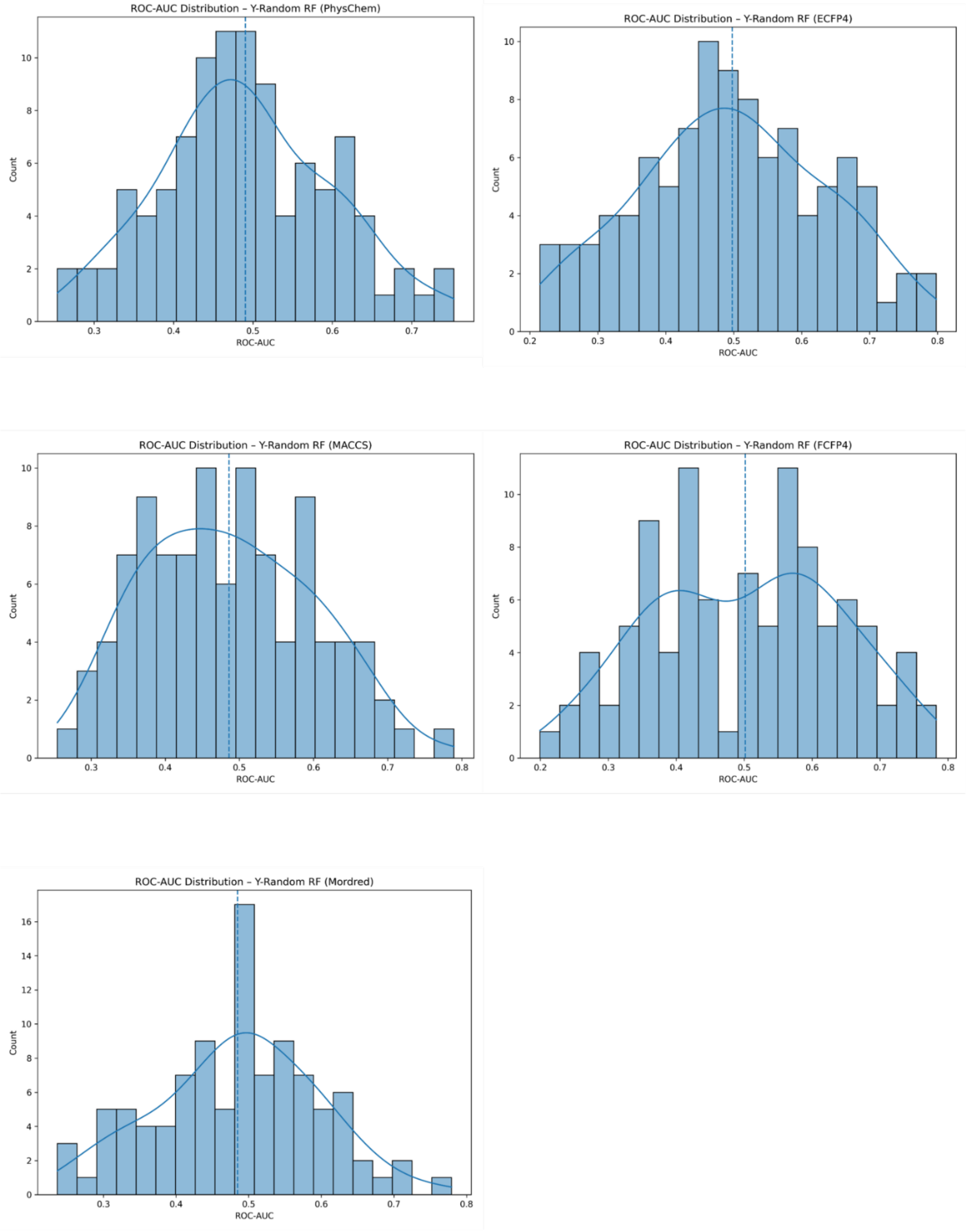

Panel B – XGBoost Algorithm

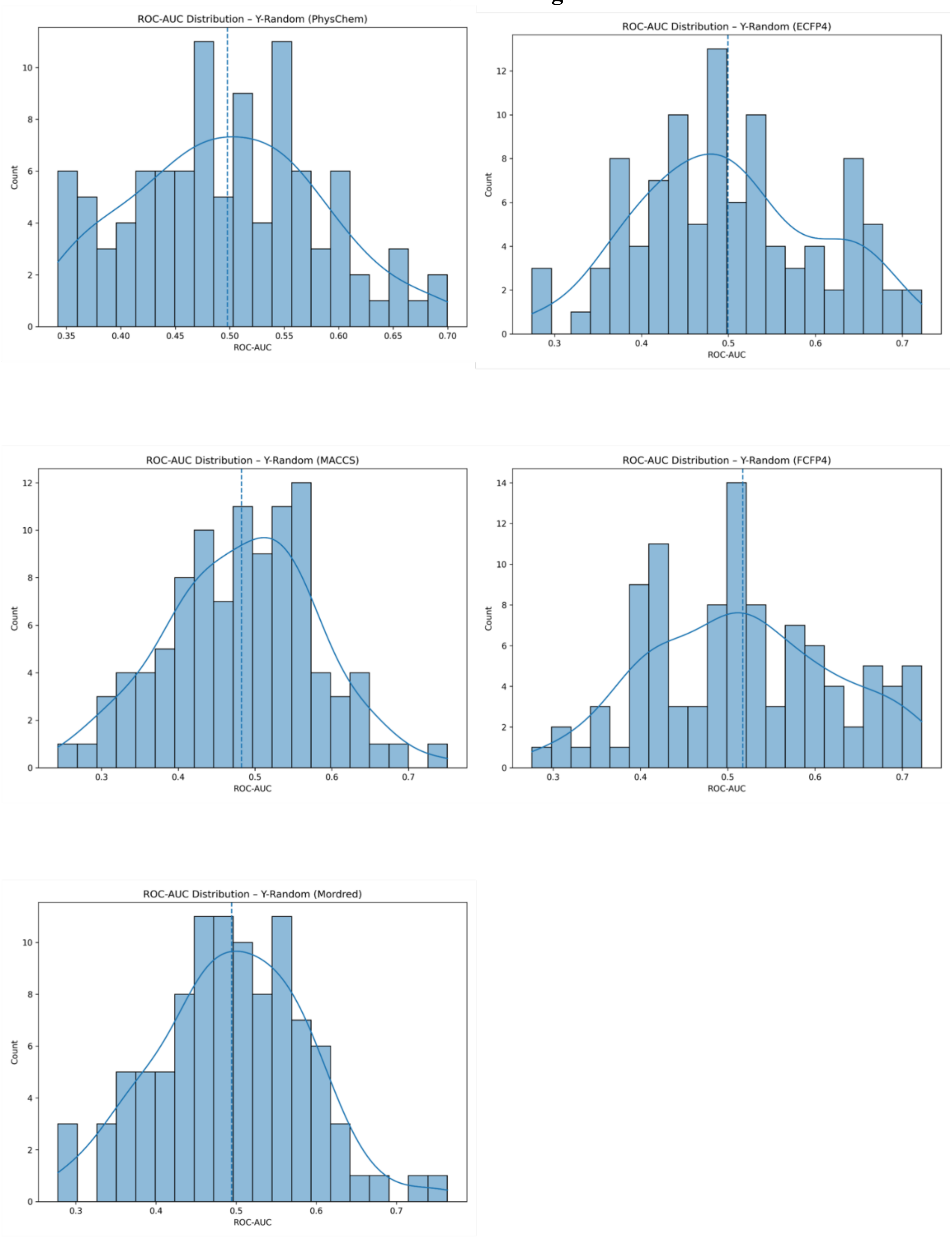

Panel C – SVM Algorithm

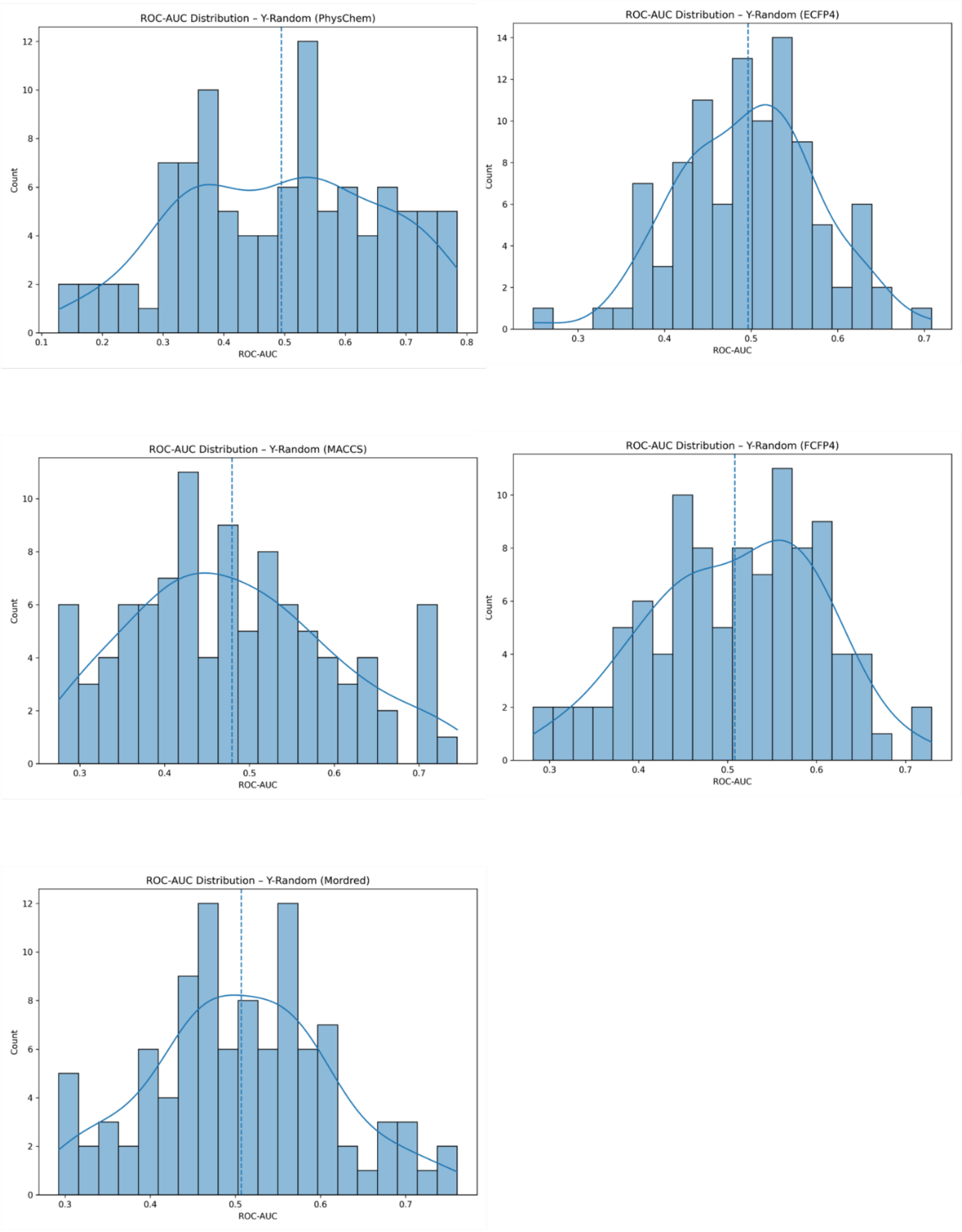

Figure S1. Distribution of the mean performance values obtained from Y-permutation tests for each QSAR model (Panels A–C).

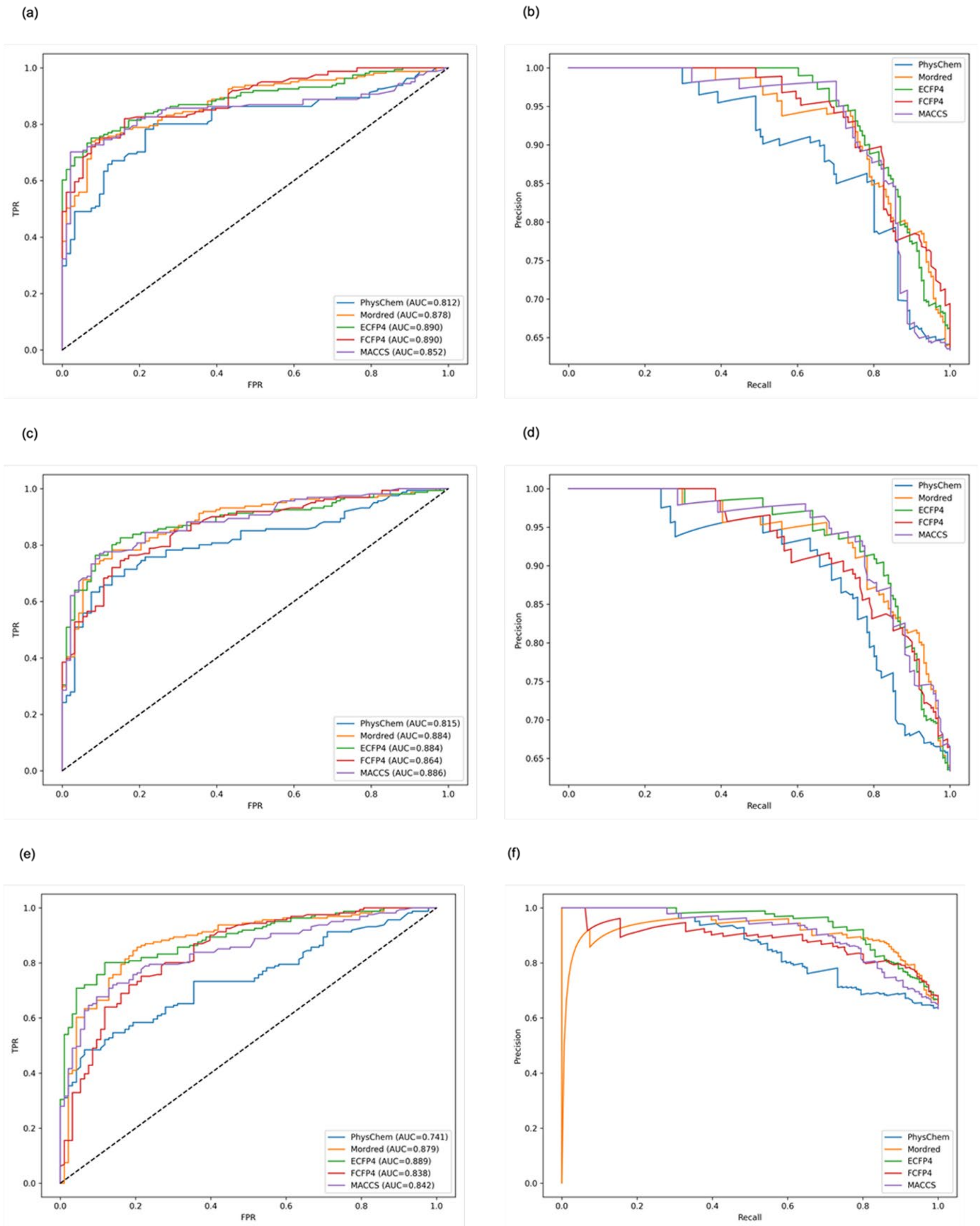

Figure S2. Receiver operating characteristic (ROC–AUC) curves (left panels) and precision–recall (PR) curves (right panels) comparing the performance of models built using different descriptor sets. Panels (a) and (b) refer to the Random Forest (RF) algorithm; panels (c) and (d) to the Extreme Gradient Boosting (XGB) algorithm; and panels (e) and (f) to the Support Vector Machine (SVM) algorithm.

Panel A – Random Forest Algorithm

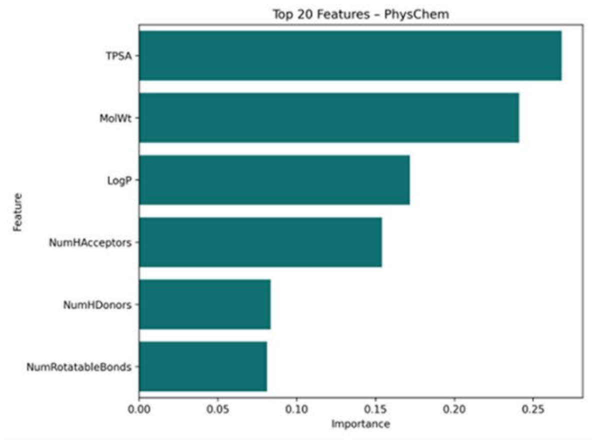

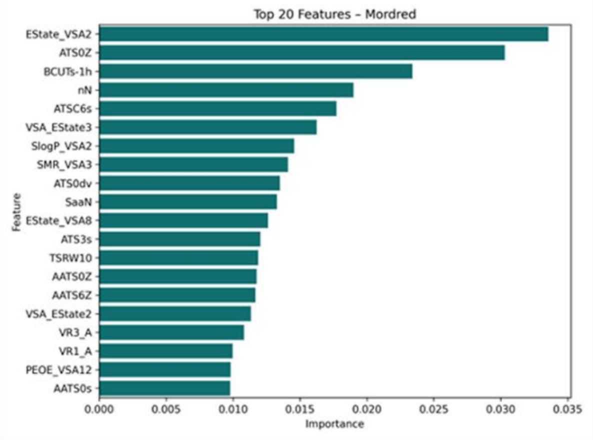

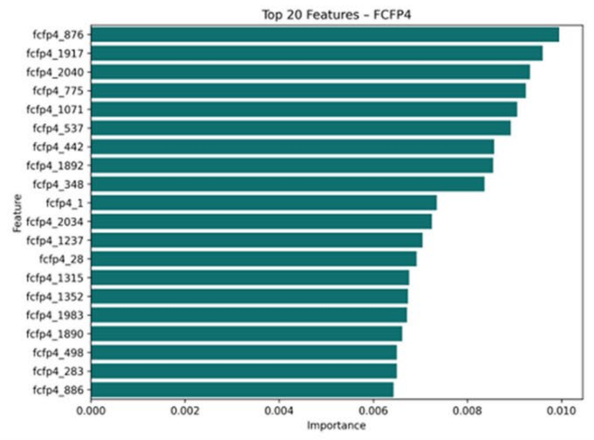

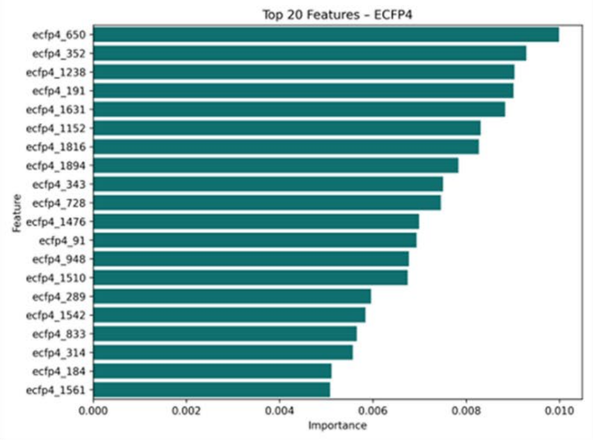

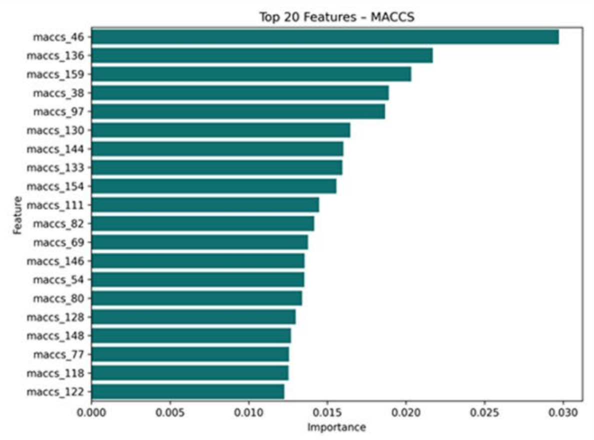

Panel B – XGBoost Algorithm

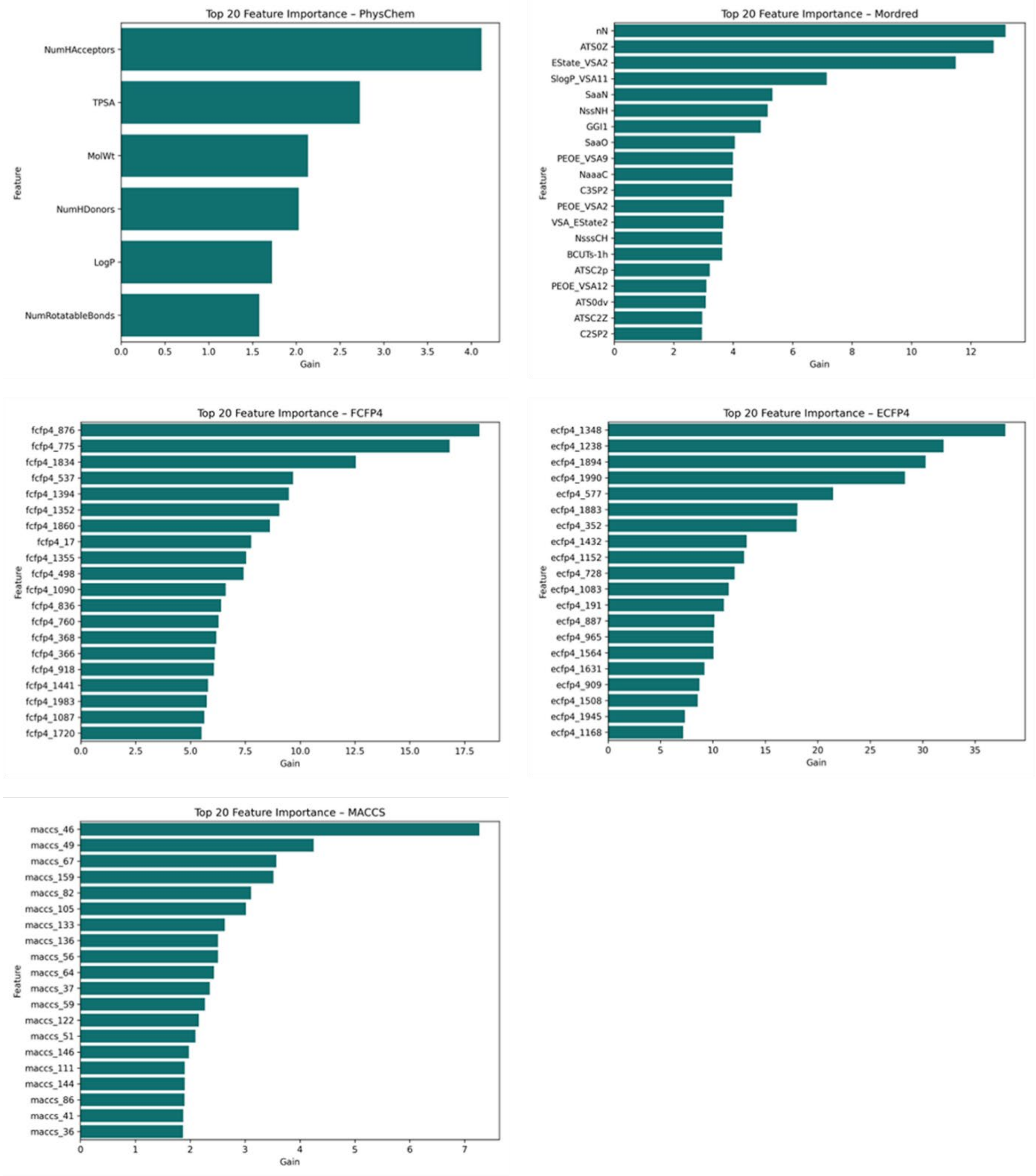

Panel C – SVM Algorithm

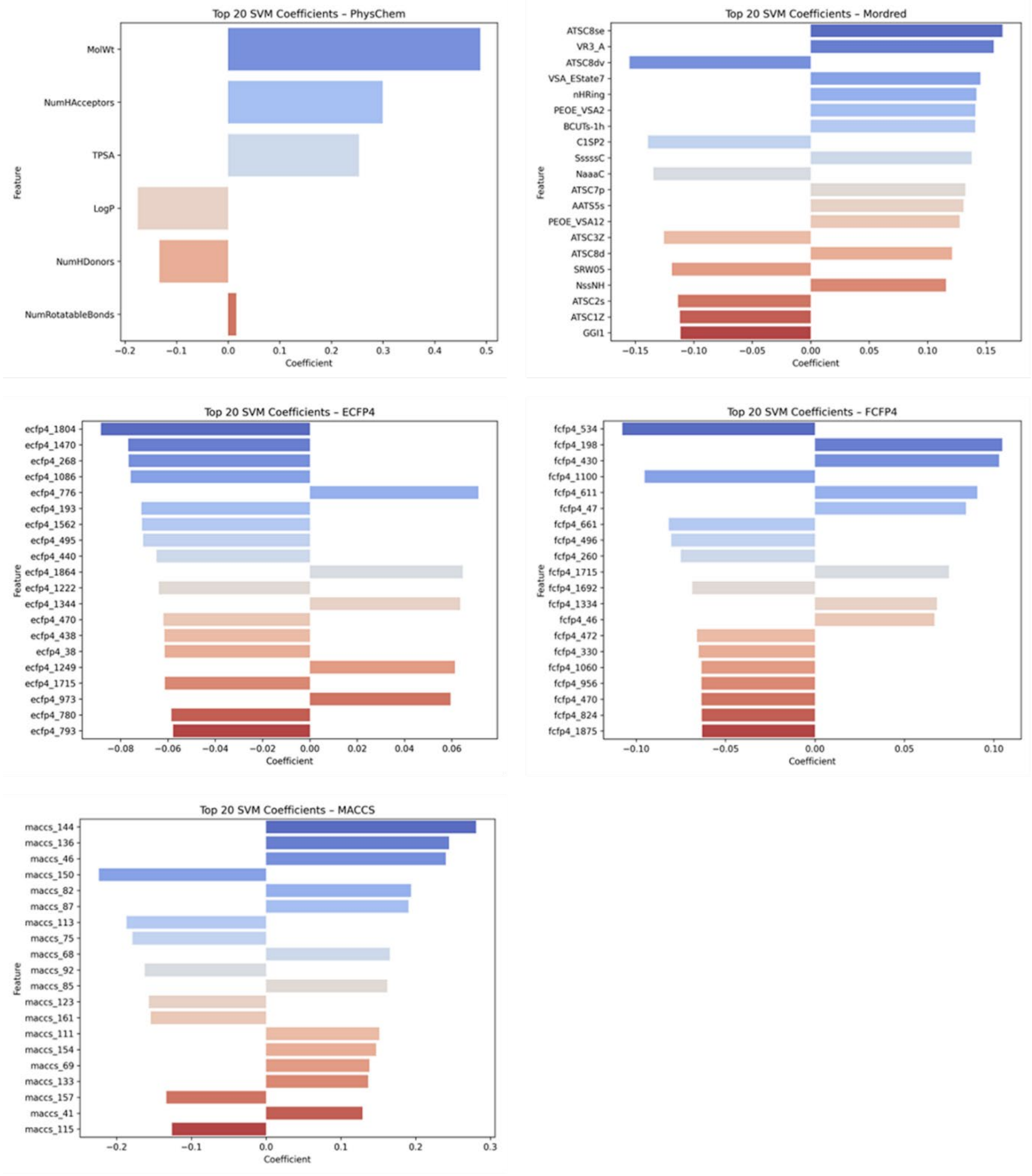

Figure S3. Feature importance profiles for each algorithm–descriptor combination. Panels (A–C) correspond to the different machine-learning algorithms evaluated.

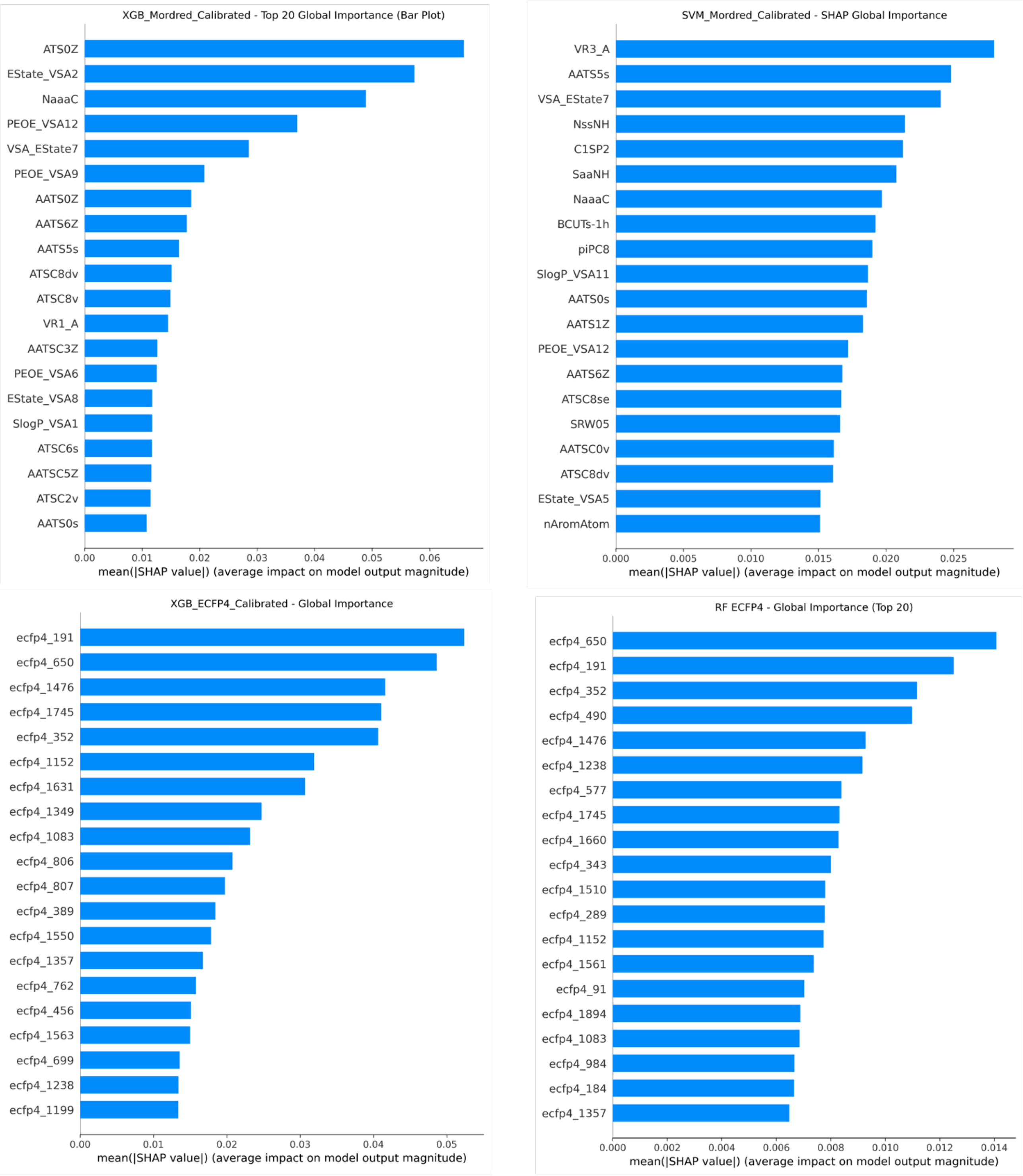

Figure S4. SHAP bar plot analysis for each algorithm–descriptor combination used in the Ensemble model.

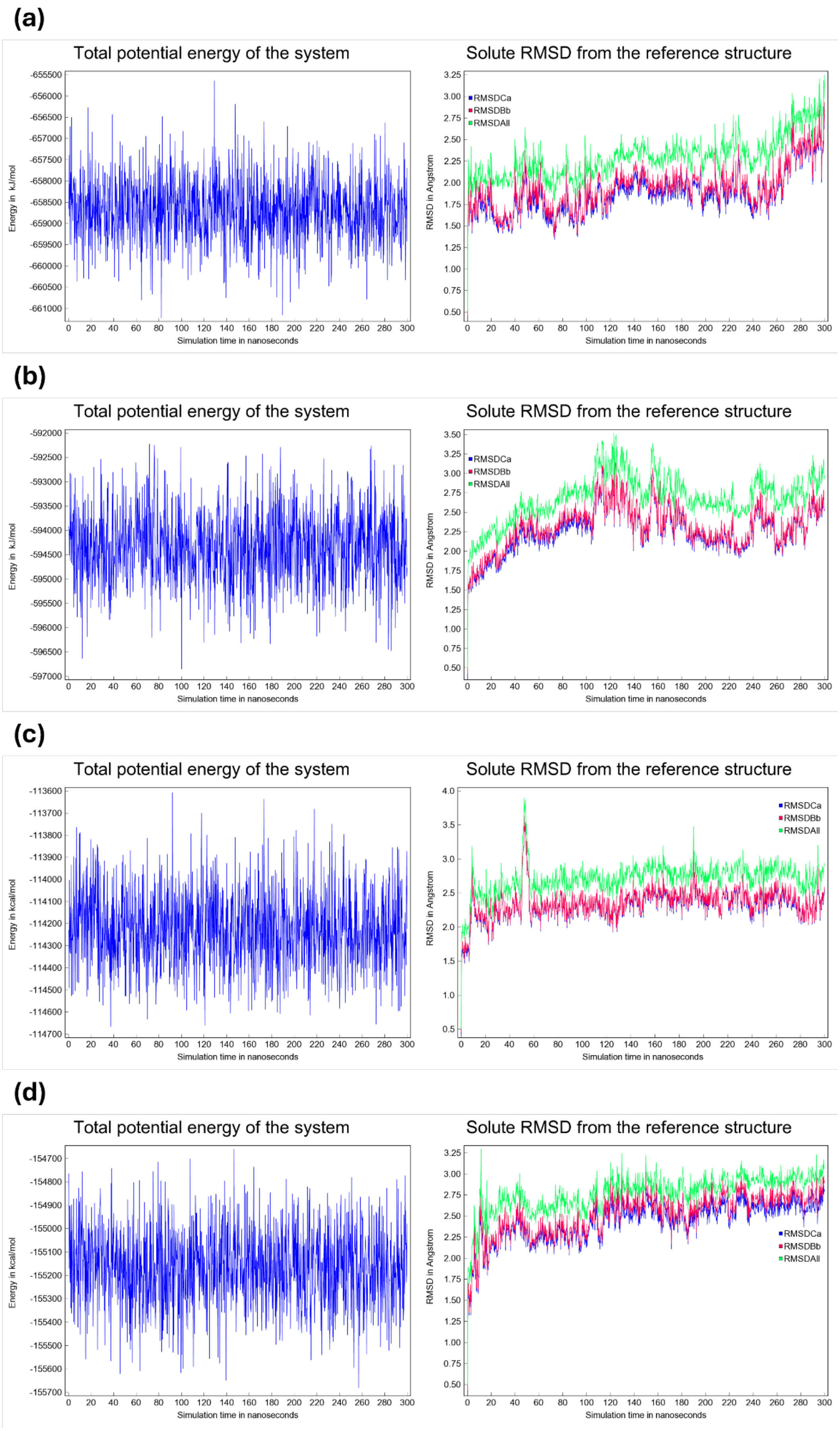

Figure S5. Left: total potential energy of the system as a function of time (300 ns) for the four ligand–protein complexes. Right: solute RMSD from the reference structure as a function of time (300 ns). Panels: (a) Ligand 6, (b) Ligand 21, (c) Ligand 25, and (d) Ligand 32.

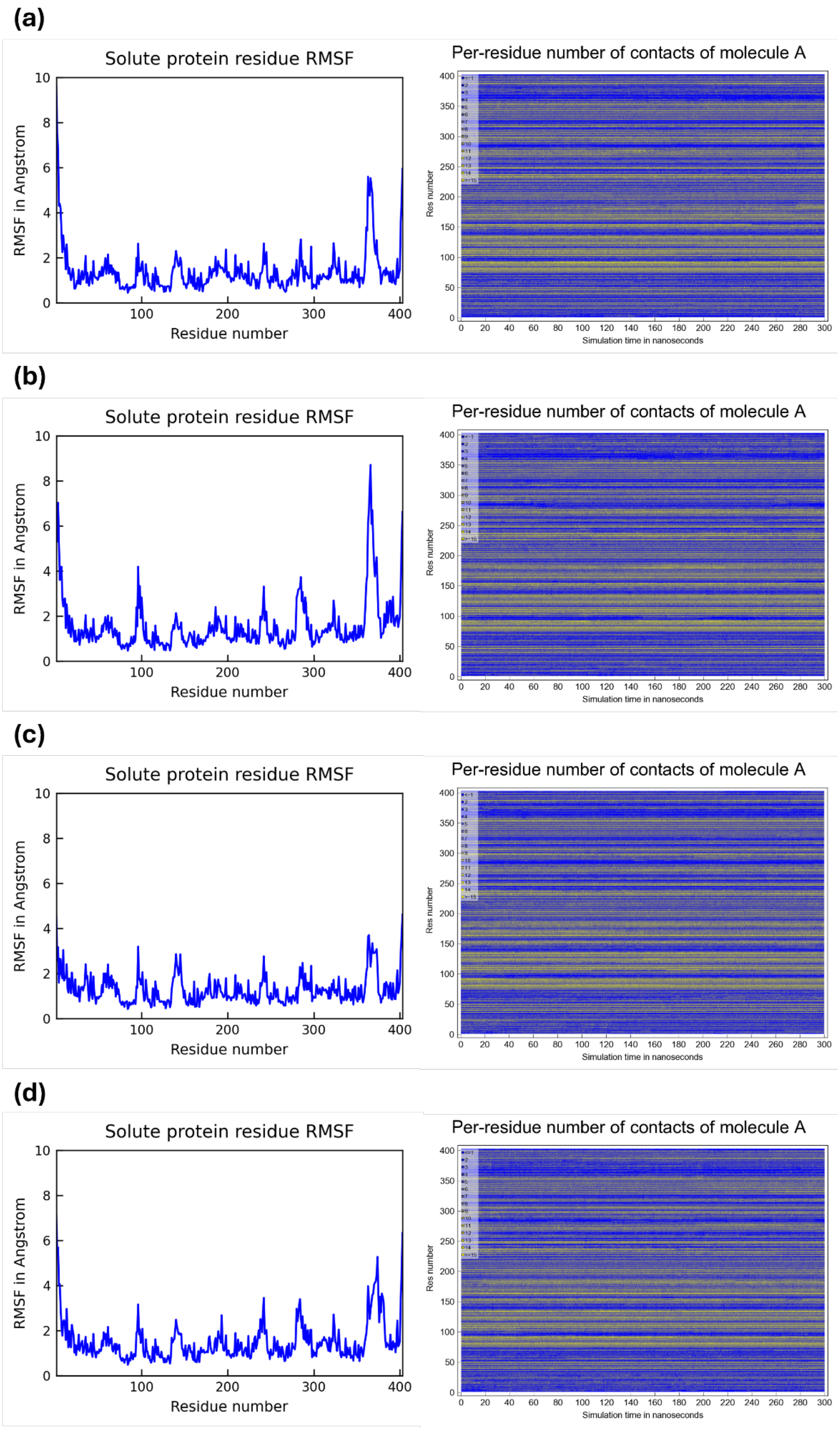

Figure S6. Left: Root-Mean-Square Fluctuation (RMSF) per solute protein residue calculated from the average RMSF of the atoms constituting the residue. Right: per-residue number of contacts as a function of simulation time for each residue number. Panels: (a) Ligand 6, (b) Ligand 21, (c) Ligand 25, and (d) Ligand 32.

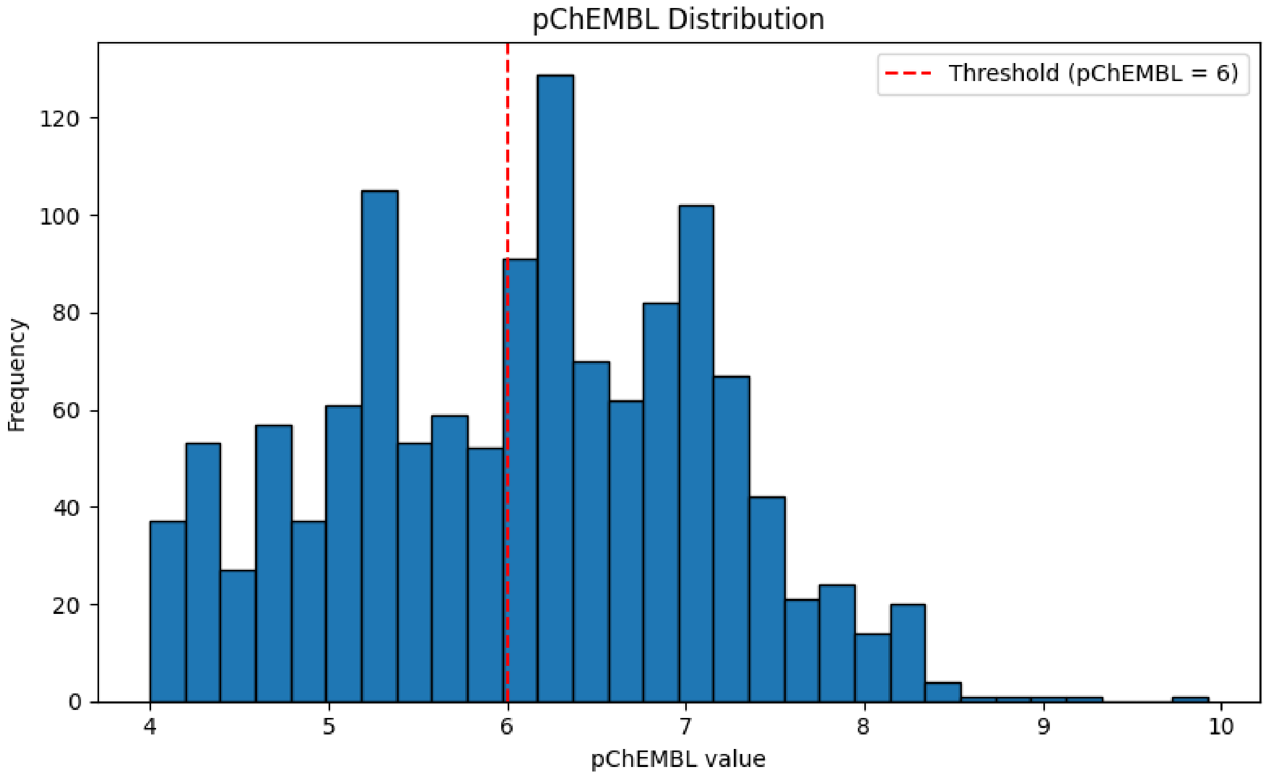

Figure S7. pCheMBL activity distribution.

Table S1. Leave-one-model-out ablation results.

Model	**ROC-AUC**	**F1**	**MCC**
Ensemble	0.895	0.846	0.638
Without_RF_ECFP4	0.893	0.827	0.624
Without_XGB_ECFP4	0.893	0.818	0.617
Without_XGB_Mordred	0.897	0.837	0.647
Without_SVM_Mordred	0.891	0.854	0.672

Table S2. Canonical SMILES codes and ensemble probability list of the FDA-approved molecules predicted as active compounds with high confidence (probability >0.7).

**Canonical SMILES**	**Predicted Probability**
O=C(NC1CCNCC1)C1CC[C@@H]2CN1C(=O)N2OS(=O)(=O)O	0.80
Cn1cnc2c(F)c(Nc3ccc(Br)cc3F)c(C(=O)NOCCO)cc21	0.79
O=C(NCc1c(F)cc(F)cc1F)c1cn2c(c(O)c1=O)C(=O)N1[C@@H]3CC[C@@H](C3)O[C@H]1C2	0.78
Cn1cnc2c(F)c(Nc3ccc(Br)cc3Cl)c(C(=O)NOCCO)cc21	0.78
CCOC(=O)C(CCc1ccccc1)NC(C)C(=O)N1C(C(=O)O)C[C@@H]2CCC[C@@H]21	0.76
CCOC(=O)C(CCc1ccccc1)NC(C)C(=O)N1C(C(=O)O)C[C@H]2CCCC[C@@H]21	0.76
N#CC1(c2ccc(NC(=O)c3cccnc3NCc3ccncc3)cc2)CCCC1	0.76
O=C(O)CCC(=O)Nc1ccc(S(=O)(=O)Nc2nccs2)cc1	0.75
C[C@@H](O)C1C(=O)N2C(C(=O)O)=C(S[C@@H]3CN[C@H](CNS(N)(=O)=O)C3)[C@H](C)[C@H]12	0.75
CN1C(C(=O)Nc2ccccn2)C(=O)c2sc(Cl)cc2S1(=O)=O	0.75
NC(CCC(=O)NC(CS)C(=O)NCC(=O)O)C(=O)O	0.75
CN1C(C(=O)Nc2ccccn2)C(=O)c2ccccc2S1(=O)=O	0.74
O=C(O)C1=C(CSc2nnnn2CS(=O)(=O)O)CS[C@H]2C(NC(=O)C(O)c3ccccc3)C(=O)N12	0.74
CCOC(=O)N[C@H]1CC[C@H]2[C@@H](CC3C(=O)O[C@@H](C)[C@@H]3[C@@H]2/C=C/c2ccc(-c3cccc(F)c3)cn2)C1	0.74
O=C(Nc1ccc([N+](=O)[O-])cc1Cl)c1cc(Cl)ccc1O	0.74
Cc1cccc(C)c1NC(=O)CN1CCCC1=O	0.73
C[C@@H]1CCO[C@H]2Cn3cc(C(=O)NCc4ccc(F)cc4F)c(=O)c(O)c3C(=O)N12	0.73
CC1CNc2c(cccc2S(=O)(=O)NC(CCCN=C(N)N)C(=O)N2CC[C@H](C)CC2C(=O)O)C1	0.73
CC1=CC(=O)c2ccccc2C1=O	0.73
O=C1c2cccc(O)c2C(=O)c2c(O)cccc21	0.73
CCOC(=O)C(CCc1ccccc1)NC(C)C(=O)N1C(=O)N(C)CC1C(=O)O	0.72
C[C@@H](O)C1C(=O)N2C(C(=O)O)=C(S[C@@H]3CNC(C(=O)Nc4cccc(C(=O)O)c4)C3)[C@H](C)[C@H]12	0.72
CN1C(C(=O)Nc2ccccn2)C(=O)c2sccc2S1(=O)=O	0.72
CN1C(=O)COc2c(C(=O)NC3CN4CCC3CC4)cc(Cl)cc21	0.72
CC(=O)c1nn(CC(=O)N2C[C@H](F)CC2C(=O)Nc2cccc(Br)n2)c2ccc(-c3cnc(C)nc3)cc12	0.71
N[C@@H]1c2cccnc2[C@H](OC(=O)N2CCC(n3c(=O)[nH]c4ncccc43)CC2)CC[C@H]1c1cccc(F)c1F	0.71
O=C(c1ccc(NS(=O)(=O)c2cccc3cccnc23)cc1)N1CCN(CC2CC2)CC1	0.71
N=C(N=C(N)NCCCCCCN=C(N)N=C(N)Nc1ccc(Cl)cc1)Nc1ccc(Cl)cc1	0.71
O=C(NCC1CN(c2ccc(N3CCOCC3=O)cc2)C(=O)O1)c1ccc(Cl)s1	0.71
Cc1nnc(SCC2=C(C(=O)O)N3C(=O)C(NC(=O)Cn4cnnn4)[C@@H]3SC2)s1	0.71
CN(CCN1CCC(OC(=O)Nc2ccccc2-c2ccccc2)CC1)C(=O)c1ccc(CN2CCC(C(N)=O)CC2)cc1	0.71
CCN1CC(C)n2c(c(O)c3c(=O)n(Cc4ccc(F)c(Cl)c4)nc(C(=O)NC)c32)C1=O	0.71
O=C1CC(=O)c2ccccc2C1=O	0.71
O=C(O)CSCC(=O)NC1CCSC1=O	0.71
NCCCCC(NC(CCc1ccccc1)C(=O)O)C(=O)N1CCCC1C(=O)O	0.71
CC(=O)NCC1CN(c2ccc(N3CCOCC3)c(F)c2)C(=O)O1	0.70
CN1CCc2nc(C(=O)N[C@H]3CC(C(=O)N(C)C)CC[C@H]3NC(=O)C(=O)Nc3ccc(Cl)cn3)sc2C1	0.70
C[C@@H]1CO[C@H]2Cn3cc(C(=O)NCc4ccc(F)cc4F)c(=O)c(O)c3C(=O)N12	0.70
NC(=O)C1CC[C@@H]2CN1C(=O)N2OS(=O)(=O)O	0.70

Table S3. Docking validation results, including RMSD, CNN score and predicted binding affinity from the redocking procedure, together with ROC–AUC and enrichment factor (EF) values derived from active/negative discrimination analysis.

PDB ID	RMSD (Å)	CNN score	CNNaffinity	ROC-AUC	EF1 %	EF5 %	EF10 %
6WJY	0.57	0.89	6.49	0.968	17.20	8.60	6.45
8ABX	0.75	0.97	6.36	0.709	17.20	4.30	4.30
9S1X	0.71	0.98	8.31	0.849	17.20	12.90	6.45

Table S4. Comparative analysis of ROC–AUC and enrichment factor (EF) values for active compound recognition obtained from individual crystal structures and ensemble consensus ranking approaches.

Method	ROC-AUC	EF5%	EF10%
6WJY	0.968	8.60	6.45
8ABX	0.709	4.30	4.30
9S1X	0.849	12.90	6.45
Best Z–score	0.946	8.60	8.60
Mean Z–score	0.901	12.90	6.45
Wheighted Z–score	0.909	12.90	6.45
Rank Consensus	0.883	12.90	6.45

Table S5. Relative MM/PBSA binding free energies (ΔG_{bind}) of the studied protein-ligand complexes. Values are expressed in kcal/mol and represent the mean ± standard deviation (std) calculated from snapshots extracted every 10 ns over the final 200 ns of the molecular dynamics' trajectories. YASARA returns binding free energy values with a positive sign, so a more positive value is considered thermodynamically more stable.

Complex	ΔG_{bind} (mean ± std)
Ligand_25	101.08 ± 34.24
Ligand_32	74.52 ± 28.34

Table S6. Grid parameters adapted for redocking performed on each cluster. Npts values for each coordinate were ever equal to 30 and spacing was ever equal to 1. The table shows the selected snapshot for each cluster and its corresponding time of simulation

Compound	Cluster	Snapshot	Time (ns)	Center x	Center y	Center z
Ligand_25	1	324	81.00	20.527	29.556	1.663
Ligand_25	2	514	128.50	20.398	30.938	6.224
Ligand_25	3	768	192.00	19.524	30.172	3.750
Ligand_25	4	931	232.75	19.188	29.357	3.864
Ligand_25	5	1094	273.50	28.184	29.823	10.202
Ligand_32	1	575	143.75	25.857	32.608	9.170
Ligand_32	2	600	150.00	26.595	31.505	8.437
Ligand_32	3	684	171.00	27.482	26.930	7.458
Ligand_32	4	732	183.00	28.511	26.457	8.211
Ligand_32	5	745	186.25	26.986	25.386	7.713
Ligand_32	6	805	201.25	29.281	28.881	14.909
Ligand_32	7	843	210.75	29.875	26.452	13.838
Ligand_32	8	1198	299.50	26.879	33.696	12.076

Table S7. Redocking performance calculated across all cluster representatives extracted from the MD trajectories. Data show the predicted affinity (CNN_VS) and the positional deviation (RMSD) of the redocked poses relative to their corresponding MD conformations of Ligand_25.

Cluster	CNN_VS	RMSD (Å)
1	9.17	0.17
2	8.57	0.26
3	8.90	0.21
4	9.02	0.20
5	8.75	0.57

Table S8. Redocking performance calculated across all cluster representatives extracted from the MD trajectories. Data show the predicted affinity (CNN_VS) and the positional deviation (RMSD) of the redocked poses relative to their corresponding MD conformations of Ligand_32.

Cluster	CNN_VS	RMSD (Å)
1	7.25	0.73
2	7.57	0.58
3	7.46	0.61
4	7.13	0.75
5	6.97	0.67
6	7.00	0.54
7	6.70	0.61
8	7.28	0.57

Table S9. Selected best hyperparameters for each algorithm-feature block. For the SVM algorithm (not shown in the table), the only parameter present, svm_C, was equal to 0.01 for all features.

Algorithm	Feature	max-depth	max-features	min_samples_leaf	Min_samples_split	n_estimators
RF	PhysChem	10	sqrt	2	2	1000
	ECFP4	None	log2	1	5	500
	FCFP4	None	log2	1	5	300
	MACCS	10	log2	1	5	300
	Mordred	20	sqrt	1	5	500
	**Feature**	**max-depth**	**colsample_bytree**	**learning_rate**	**subsample**	**n_estimators**
XGB	PhysChem	4	0.7	0.05	0.7	300
	ECFP4	4	0.6	0.05	0.8	300
	FCFP4	6	0.6	0.05	0.7	300
	MACCS	6	0.6	0.05	0.6	300
	Mordred	6	0.7	0.07	0.8	300

Table S10. Grid box parameters used for each crystal structure in docking simulations. The spacing setting was always equal to 1.

Model	Center x	Center y	Center z	Npts x	Npts y	Npts z
6WJY	61.175	51.418	22.177	24	24	32
8ABX	18.546	37.843	18.196	24	32	24
9S1X	20.137	16.464	4.559	22	32	26
